# GDE2 is essential for neuronal survival in the postnatal mammalian spinal cord

**DOI:** 10.1186/s13024-017-0148-1

**Published:** 2017-01-19

**Authors:** Clinton Cave, Sungjin Park, Marianeli Rodriguez, Mai Nakamura, Ahmet Hoke, Mikhail Pletnikov, Shanthini Sockanathan

**Affiliations:** 1 The Solomon H. Snyder Department of Neuroscience, Johns Hopkins University School of Medicine, 725 N Wolfe Street, PCTB 1004, Baltimore, MD 21205 USA; 2 Department of Neurology, Johns Hopkins University School of Medicine, Baltimore, USA; 3 Department of Psychiatry, Johns Hopkins University School of Medicine, Baltimore, USA; 4 University of Utah, BPRB 390D South 2030 East, Salt Lake City, UT 84112 USA; 5 Bascom Palmer Eye Institute, 900 NW 17th St, Miami, FL 33136 USA

**Keywords:** GDE2, Motor neuron, Neurodegeneration, GPI-Anchor

## Abstract

**Background:**

Glycerophosphodiester phosphodiesterase 2 (GDE2) is a six-transmembrane protein that cleaves glycosylphosphatidylinositol (GPI) anchors to regulate GPI-anchored protein activity at the cell surface. In the developing spinal cord, GDE2 utilizes its enzymatic function to regulate the production of specific classes of motor neurons and interneurons; however, GDE2’s roles beyond embryonic neurogenesis have yet to be defined.

**Method:**

Using a panel of histological, immunohistochemical, electrophysiological, behavioral, and biochemistry techniques, we characterized the postnatal *Gde2* ^−/−^ mouse for evidence of degenerative neuropathology. A conditional deletion of *Gde2* was used to study the temporal requirements for GDE2 in neuronal survival. Biochemical approaches identified deficits in the processing of GPI-anchored GDE2 substrates in the *SOD1* ^G93A^ mouse model of familial Amyotrophic Lateral Sclerosis that shows robust motor neuron degeneration.

**Results:**

Here we show that GDE2 expression continues postnatally, and adult mice lacking GDE2 exhibit a slow, progressive neuronal degeneration with pathologies similar to human neurodegenerative disease. Early phenotypes include vacuolization, microgliosis, cytoskeletal accumulation, and lipofuscin deposition followed by astrogliosis and cell death. Remaining motor neurons exhibit peripheral motor unit restructuring causing behavioral motor deficits. Genetic ablation of GDE2 after embryonic neurogenesis is complete still elicits degenerative pathology, signifying that GDE2’s requirement for neuronal survival is distinct from its involvement in neuronal differentiation. Unbiased screens identify impaired processing of Glypican 4 and 6 in *Gde2* null animals, and Glypican release is markedly reduced in *SOD1* ^G93A^ mice.

**Conclusions:**

This study identifies a novel function for GDE2 in neuronal survival and implicates deregulated GPI-anchored protein activity in pathways mediating neurodegeneration. These findings provide new molecular insight for neuropathologies found in multiple disease settings, and raise the possibility of GDE2 hypofunctionality as a component of neurodegenerative disease.

**Electronic supplementary material:**

The online version of this article (doi:10.1186/s13024-017-0148-1) contains supplementary material, which is available to authorized users.

## Background

Throughout life, neurons must contend with a multitude of stresses both intrinsic and extrinsic. Supplied with only a finite number from birth, and excluding a few notable neurogenic niches [1, 2], the adult nervous system has no method for the bulk replacement of a neuronal population. As such, the nervous system must employ distinct mechanisms to actively ensure neuronal health and survival. What these mechanisms are and how they integrate with cellular pathways are not well understood. Moreover, identifying proteins responsible for neuronal survival is crucial to understand the multifactorial pathogenesis of human neurodegenerative disease.

The six-transmembrane glycerophosphodiester phosphodiesterases (GDEs) are a small family of three proteins that are related to a larger group of one-pass, two-pass and secreted proteins through common possession of an enzymatic domain homologous to bacterial glycerophosphodiester phosphodiesterase (GDPD) [3, 4]. Six-transmembrane GDEs consist of GDE2, GDE3 and GDE6, and their enzymatic GDPD domains face the extracellular space [3, 5]. During embryogenesis, GDE2, also known as GDPD5, is expressed in postmitotic neurons in the spinal cord and the brain. Functional studies in the mouse and chick demonstrate that GDE2 is necessary and sufficient for motor neuron generation. In the developing chick spinal cord, overexpression of GDE2 in motor neuron progenitors drives their premature differentiation into postmitotic motor neurons, while ablating GDE2 by RNA interference profoundly reduces motor neuron differentiation [6, 7]. GDE2 plays analogous roles in mammalian neurodevelopment. *Gde2* knock out (KO) mice fail to generate specific subsets of late born alpha motor neurons as well as distinct populations of spinal interneurons [8, 9]. In the brain, GDE2 loss leads to a decrease in deep layer neurons but an expansion of upper layer neurons [10]. Importantly, point mutations to critical residues within the enzymatic GDPD domain abolish GDE2’s ability to promote differentiation, demonstrating the central role of extracellular enzymatic activity to GDE2 function [6].

Glycosylphosphatidylinositol (GPI)-anchorage is a common post-translational modification employed to adhere proteins to the extracellular leaflet of the plasma membrane via covalent attachment of a lipid moiety to the protein’s C-terminus [11]. Approximately 10–20% of all cell surface protein is GPI-anchored while over 150 unique mammalian proteins have known GPI-anchorages [12]. Activities of soluble versus membrane-tethered GPI-anchored proteins differ, implying a critical role for pathways that release GPI-anchored proteins from the cell membrane. We have shown that GDE2 possesses the unique capacity to cleave within the GPI-anchor of proteins tethered to the cell surface. During motor neuron differentiation, GDE2 functionally inactivates RECK (REversion-inducing Cysteine-rich protein with Kazal motifs) by cleaving its GPI-anchor, releasing it from the cell surface of motor neurons. This leads to a non-cell autonomous decrease in Notch signaling in neighboring progenitors, promoting cell-cycle exit and differentiation. Similarly, GDE2 mediated cleavage of Glypican 6 at the cell surface has recently been reported to promote the differentiation of neuroblastoma cells [13]. GPI-anchor cleaving activities were also detected in GDE3 and GDE6, providing evidence that the six-transmembrane GDEs are the first known GPI-anchor cleaving enzymes in mammals that function at the cell surface [9]. Interestingly, a number of GPI-anchored proteins have been implicated in neurodegenerative disease. For example, conversion of GPI-anchored cellular prion protein into cytotoxic aggregates is a main focus in Creutzfeld-Jacob disease [14]. Expression of GPI-anchored CD59 has been shown to be neuroprotective in neurons and decreased expression has been reported in the brains of patients with Alzheimer’s Disease (AD) [15], and aberrant Glypican processing has been detected in the context of AD and Niemann-Pick Disease [16, 17]. In Amyotrophic Lateral Sclerosis (ALS), expression of GPI-anchored Ephrin A5 and uPAR are both significantly altered [18, 19]. These observations underscore the importance of GPI-anchor regulation in disease, and open an avenue for GDE2 dysfunction to mediate aspects of neurodegeneration.

Here, we find that aged *Gde2* KO mice develop a protracted, progressive neurodegeneration afflicting spinal motor neurons that resembles pathologies frequently observed across human neurodegenerative settings [20, 21]. Timed ablation of GDE2 after motor neuron differentiation is complete elicits neurodegeneration in adult animals, indicating that GDE2 function in neuronal survival is distinct from its role in embryonic neurogenesis. In addition, we observe the reduced release of Glypican proteins that are known substrates of GDE2 in *SOD1* ^G93A^ animals, which exhibit profound motor neuron degeneration. This study identifies GDE2 as an important regulator of neuronal survival in the adult nervous system, and invokes the possibility that deregulated GPI-anchored substrate activities are components of motor neuron degenerative pathways.

## Methods

### Animal husbandry

Mice were maintained and used in accordance with approved Johns Hopkins University IACUC protocols. *Gde2* ^+/−^ and *Gde2* ^lox/+^ mice with mixed backgrounds (129/SV x C57BL/6J) were out-crossed to C57BL/6J (Jackson Laboratories 000664) for four generations. *Gde2* ^lox/+^;*ROSA*:Cre^ER^mice were maintained on a C57BL/6J background and crossed with *Gde2* ^−/−^ for conditional ablation of *Gde2*. B6SJL-Tg(SOD1*G93A)1Gur/J mice were obtained from Jackson Laboratory (Stock# 002726), and maintained as a hemizygous line on a C57BL/6J background. Animals for 6 month motor neuron counts were pooled from 6 to 7 month old animals. Genotyping primers, competitive PCR analysis, and 4-OHT administration are as previously described [8], and B6SJL-Tg(SOD1*G93A)1Gur/J mice were genotyped as specified by Jackson Laboratory.

### Tissue preparation

Animals were anesthetized with Avertin solution (1.3% 2,2,2-Tribromorethanol (Fluka 90710) and 0.7% 2-methyl-2-butanol (Sigma 240486) in Phosphate Buffered Saline (PBS)) delivered via intraperitoneal injection at 0.02 ml/g body weight. After confirming the absence of a pain reflex, animals were transcardially perfused with 0.1 M Phosphate Buffer (PB) followed by 4% paraformaldehyde (PFA) in 0.1 M PB. Regions of interest were dissected and processed into cyromolds or paraffin blocks. *Cryosections*: Tissue samples were post-fixed in 4% PFA for 1 h at 4 °C, rinsed in PBS, and incubated in 30% sucrose for 12–24 h. Samples were embedded in O.C.T Compound (Tissue-Tek 62550–12) and flash frozen in a dry ice ethanol bath. Cryomolds were stored at −80 °C and sectioned on an UltraPro 5000 Cryostat (Vibratome). *Paraffin Sections:* Tissue samples were post-fixed in 4% PFA for 12–15 h at 4 °C, rinsed in PBS, and incubated in Decalcifying Solution-Lite (Sigma D0818-1L) for 7 h at room temperature. Samples were then dehydrated in a graded ethanol series and cleared in Xylenes (Fisher Scientific X3P-1GAL). Samples were then infiltrated in Blue Ribbon Paraffin wax (Leica Microsystems 3801360) for 12–15 h and embedded. Sections were cut with a Rotary Microtome (Leica RM2235). For staining, slides were deparaffinized in Xylenes (Fisher Scientific X3P-1GAL), and rehydrated in an ethanol series. After staining, fluorescent slides were dehydrated in ethanol and coverslipped with Vectashield mounting medium (Vector Laboratories H1000); pathology slides were cleared with Xylenes, and coverslipped with Permount (Fisher Scientific SP15-500).

### Pathology stains

*Hematoxylin and Eosin:* Sections were incubated in Harris Hematoxylin (VWR 95057–858) for 8 min, differentiated in 1% acid alcohol for 4 s, and incubated in Bluing Reagent Solution (VWR 95057–852) for 2 min. Sections were then counterstained with Eosin-phloxine solution (VWR 95057–846). For quantification, a vacuolated neuron was defined as having 2 or more clear somal abscesses greater than 2 μm in diameter. A minimum of 200 neurons were analyzed per animal. *Sudan Black B*: Sections were incubated in 1% Sudan Black B (Sigma 199664-25G) in 70% ethanol for 2 min. Kohler illuminated brightfield images were acquired on an Axioskop 2 (Zeiss) with a Retiga 2000R CCD camera (QImaging). Brightness and contrast were adjusted equally between experimental conditions. Post-hoc color balance was adjusted to achieve uniform white balance on all images.

### Fluorescent *in-situ* hybridization

In-situ hybridizations were performed with modifications from Schaeren-Wiemers and Gerfin-Moser, 1993 [22]. Briefly, endogenous peroxidase activity was blocked with 0.3% hydrogen peroxide + 0.1% sodium azide. Sections were acetylated in 0.5% acetic anhydride and permeabilized with 0.1% Triton X-100 (Sigma T8787). Slides were hybridized with digoxigenin-labeled probe overnight at 65 °C in an RNAse free chamber. 680 bp sense and antisense probes were generated from the 3′ UTR of mouse *Gde2.* After hybridization, sections were washed with 50% formamide/sodium citrate buffers at 65 °C. Sections were treated with blocking buffer (Roche 11096176001) and incubated with sheep anti-digoxigenin-POD, 1:250 (Roche 11207733910) overnight at 4 °C. Fluorescent signal was developed with the Tyramide Signal Amplification Kit (PerkinElmer NEL741001KT) according to the manufacturer’s instructions.

### Immunohistochemistry

Sections were washed in Phosphate Buffered Saline + 0.3% Triton X-100 (PBST) and microwave boiled in 10 mM sodium citrate, pH 6.0 for 10 min for antigen retrieval. Slides were blocked with 5% bovine serum albumin and incubated with primary antibodies overnight at 4 °C. Primary antibodies were visualized with fluorescently conjugated secondary antibodies (Jackson Immunoresearch). Whole mount muscle staining was performed without antigen retrieval. Dissected muscle was incubated in primary antibody + Alexa 488 conjugated α-Bungarotoxin (Invitrogen B13422) for 24 h at room temperature and incubated in secondary antibody for 48 h at room temperature while rotating. Confocal images were taken on a Zeiss LSM 700 with identical acquisition parameters between experimental groups. Any image contrast and brightness adjustments were made equally between experimental groups. GFAP area fraction was calculated in ImageJ by thresholding regions of interest (ROI) above background and dividing the area of the GFAP^+^ processes by the total ROI area (150 μm^2^). Peripherin intensity measurements were taken in ImageJ using a 7.5 μm^2^ ROI centered over the cell soma. Antibodies used in this study: rabbit anti-GDE2, 1:1000 (Covance); goat anti-Choline Acetyltransferase, 1:100 (Millipore AB144P); mouse anti-NeuN, 1:200 (Millipore MAB377); mouse anti-GFAP, 1:500 (BD Pharmingen 556328); rabbit anti-Neurofilament-H, 1:500 (Millipore AB1989); rabbit anti-Peripherin, 1:400 (Millipore AB1530); rabbit anti-Iba1, 1:500 (Wako 019–19741); rat anti-Lamp2, 1:500 (DSHB ABL-93); rat anti-CathepsinD, 1:500 (R&D Systems MAB1029).

### Motor neuron quantification

Motor neurons were quantified from paraffin sections of lumbar (L3-L5) spinal cord. Alpha (ChAT^+^/NeuN^+^) and gamma (ChAT^+^/NeuN^−^) motor neuron numbers were averaged from a minimum of 15 sections and a minimum of 140 neurons per animal to ensure accurate sampling of heterogeneously distributed motor neurons. Total neurons counted per time point: 13 months: control = 2249, *Gde2* KO = 1573; 6 months: control = 1674, *Gde2* KO = 950. The LMC was defined as all neurons in the lateral aspect of spinal lamina 7. The MMC was defined as all neurons in the medial portion of spinal lamina 8.

### Lipofuscin imaging

Autofluorescent lipofuscin particles were imaged in unstained paraffin sections on a Zeiss LSM 700 using a 405 nm excitation laser and a long-pass 505 nm emission filter. Lipofuscin puncta were quantified from a single, in-focus 0.7 μm z-plane from neurons in the ventral horn. Lipofuscin spectra were measured from in-situ deposits in paraffin embedded spinal cord sections using a Zeiss LSM 510 equipped with a meta-detector and a near-infrared tunable pulsed femtosecond Ti:Sapphire laser (Chameleon Ultra II). Using 2-photon excitation at 770 nm, the emitted light was quantified in 10 nm increments from 367 nm to 699 nm with the meta-detector from the region of interest. The excitation spectrum was estimated by varying the 2-photon wavelength from 690 nm to 870 nm and measuring intensity of the emitted light filtered through a 500–550 nm band pass filter. The average intensity was then plotted using the approximate 1P wavelength (roughly λ/2).

### Biochemistry

*Lysate Preparation*: Freshly dissected lumbar spinal cord segments were lysed and separated into total and membrane fractions as previously described to detect Neurofilament and GDE2, respectively [9]. *Sequential Solubilization:* Dissected lumbar spinal segments were first homogenized in Tris Buffer (10 mM Tris, 150 mM NaCl, pH 7.5) for 15 s with a Pellet Pestle (Kimble 749540). Samples were centrifuged 5000 x G for 5 min. Supernatant (S1 fraction) was transferred to a new tube. The insoluble pellet was homogenized in Tris Buffer + 60 mM Octyl β-D-glucopyranoside (Sigma O8001) for 15 s with a Pellet Pestle. Samples were centrifuged at 3000 x G for 5 min. Supernatant (S2 fraction) was transferred to a new tube. *Alpha Toxin Pull Down Assay*: Alpha toxin from *Clostridium septicum* was expressed and purified as previously described [23] with the following modifications. An N-terminal HaloTag + C-terminal histidine tagged alpha toxin plasmid was transformed in Rosetta (DE3)pLysS *Escherichia coli*. Bacterial pellets were then resuspended in HaloTag protein purification buffer (50 mM HEPES, 150 mM NaCl, 1 mM DTT, 0.5 mM EDTA, and 0.005% IGEPAL CA-630), supplemented with 0.2 mg/mL lysozyme, 0.5 mg/mL DNase I. Cells were lysed with repeated rounds of freeze-thaw and centrifuged at 10,000 x G for 15 min at 4 °C. The supernatant was incubated with TALON metal affinity resin (Clontech) overnight at 4 °C. Alpha toxin was eluted in 50 mM PB, 300 mM NaCl, 150 mM imidazole, and the eluted sample was dialyzed and incubated with HaloLink resin for 1 h at room temperature. Proteins were then cross-linked with 0.05 mg/mL DSS. Soluble protein extract from WT and *Gde2* KO mice were incubated with the cross-linked alpha toxin overnight at 4 °C, and captured proteins were eluted from the alpha toxin using 2% SDS. *Western Blot*: Samples were run on 10% SDS-PAGE in Tris-glycine buffer. Proteins were transferred to PVDF membrane, washed with TBST, blocked with 5% milk, and probed with the desired antibodies: rabbit anti-GDE2 (Convance 1:1000), rabbit anti-Neurofilament H (Millipore AB1989), mouse anti-Actin (Millipore MAB1501R); rabbit anti-Glypican 1 (Abcam AB55971), rabbit anti-Glypican 4 (Immundiagnostik AH1007.2), goat anti-Glypican 6 (R and D Systems AF1053), rabbit anti-Na/K ATPase (Abcam AB76020), rabbit anti-GAPDH (Cell Signaling 8884). Blots were developed using HRP-conjugated secondary antibodies (Jackson Immunoresearch) with a chemiluminescent substrate. Densitometry measurements were made with ImageJ.

### Mass spectrometry

Isolated gel pieces were subjected to a modified in-gel trypsin digestion procedure [24]. Gel pieces were dehydrated with acetonitrile for 10 min and then completely dried in a speed-vac. Gel pieces were rehydrated with 50 mM ammonium bicarbonate solution containing 12.5 ng/μl modified sequencing-grade trypsin (Promega) at 4 °C. The excess trypsin solution was removed and replaced with 50 mM ammonium bicarbonate solution, and samples were then placed in a 37 °C room overnight. Peptides were later extracted by removing the ammonium bicarbonate solution, followed by one wash with a solution containing 50% acetonitrile and 1% formic acid and dried. Samples were reconstituted in 5–10 μl of HPLC solvent A (2.5% acetonitrile, 0.1% formic acid). A nano-scale reverse-phase HPLC capillary column was created by packing 2.6 μm C18 spherical silica beads into a fused silica capillary (100 μm inner diameter x ~30 cm length) with a flame-drawn tip [25]. After equilibrating the column each sample was loaded via a Famos auto sampler (LC Packings) onto the column. A gradient was formed and peptides were eluted with increasing concentrations of solvent B (97.5% acetonitrile, 0.1% formic acid). As peptides eluted they were subjected to electrospray ionization and then entered into an LTQ Orbitrap Velos Pro ion-trap mass spectrometer (Thermo Fisher Scientific). Peptides were detected, isolated, and fragmented to produce a tandem mass spectrum of specific fragment ions for each peptide. Peptide sequences were determined by matching protein databases with the acquired fragmentation pattern using Sequest (Thermo Fisher Scientific) [26]. All databases include a reversed version of all the sequences and the data was filtered to between a one and two percent peptide false discovery rate.

### Electrophysiology

Animals were anesthetized under isofluorane anesthesia and body temperature maintained at 37 °C with a heating pad. A subdermal ground electrode was positioned in the upper back. Recordings were made with a PowerLab signal acquisition platform (ADInstruments). *Motor Nerve Conduction Study (MNCS)*: Compound Motor Action Potentials (CMAPs) were obtained from the animal’s right hindpaw. Platinum subdermal needle electrodes (Grass Instruments) were placed in plantar foot pads and stimulating electrodes were placed into the sciatic notch. The average of 5 CMAPs evoked with supramaximal stimulation was recorded. *Motor Unit Number Estimation*: The lower hindlimb was fitted with a circumferential surface electrode (CareFusion 019–439300) lined with electroconductive gel (Parker Laboratories 15–60). Sequentially increasing stimulation was delivered with needle electrodes positioned in the sciatic notch until discrete CMAPS were observed. The first 10 stable motor units were recorded per animal followed by the supramaximal CMAP. Both hindlimbs of each animal were tested. *Sensory Nerve Conduction Study (SNCS)*: Sensory Nerve Action Potentials (SNAPs) were recorded by placing needle electrodes in the dorsal aspect of the proximal tail and stimulating 5 cm distally. SNAP width is the time required for the SNAP waveform to return to baseline, and conduction velocity is calculated by dividing the recording distance (5 cm) by the distal latency.

### Behavior

Mouse behavioral tests were performed at the Johns Hopkins University Brain Science Institute Behavioral Core. Male mice (10 WT and 10 *Gde2* KO) were followed longitudinally with evaluation at 6 months and 13 months. Tests were performed sequentially; first grip strength, then open field followed by heat sensitization. Each test was done on a different day to reduce animal stress and fatigue. Electrophysiological experiments (described above) were performed after the behavior at each time point. To minimize variability, tests were only performed by 1 person between the hours of 11 am and 4 pm in the light cycle. WT and KO animals were housed and tested together with unrestricted access to food and water. Prior to the first test at each time point, animals were handled by the experimenter for one hour on 5 consecutive days. *Open Field*: Individual animals were recorded for 30 min while they explored an open field activity chamber (Photobeam Activity System, San Diego Instruments). Movement was measured as disruptions in a grid of infrared beams lining the base of the chamber. During the recording, the chambers were enclosed in ventilated, light-proof cabinets. *Grip Strength*: Grip strength was measured by lifting the animal by the tail so its forelimbs or hindlimbs can grab the grid of the grip strength meter (Grip Strength Test, IITC Life Science). When the animal is withdrawn, the force of the grip is recorded by a force transducer. In a session, each animal is tested 5 times with at least a 30 min rest in the home cage in between measurements. *Heat Sensitization*: Animals are habituated in clear plexiglass enclosures resting on an elevated glass platform heated to 32 °C (Plantar Test Analgesia Meter 390G, IITC Life Science). Latency is recorded as the interval between activating a heat lamp under the rear right paw and withdrawal of the paw from the targeted position. Per session, each animal is tested 7 times with at least 30 min of rest on the platform between measurements.

### Statistical analysis

All graphs represent mean ± SEM. Longitudinal behavior experiments compared across multiple time points were analyzed with a two-way Analysis of Variance (ANOVA) with repeated measures. All other quantitative data was analyzed with an unpaired Student’s t-test. Significance level is a value of *p* ≤ 0.05. Cell counts in Fig. 6e, h, and Additional file 1: Figure S2 are all normalized to the mean of the control values for clarity of presentation. Statistical analysis was performed on the raw data. The n-numbers as stated in the figure legends refer to individual animals processed and analyzed equivalently between the experimental groups.

## Results

### GDE2 is expressed in the postnatal spinal cord

**Fig. 1:**
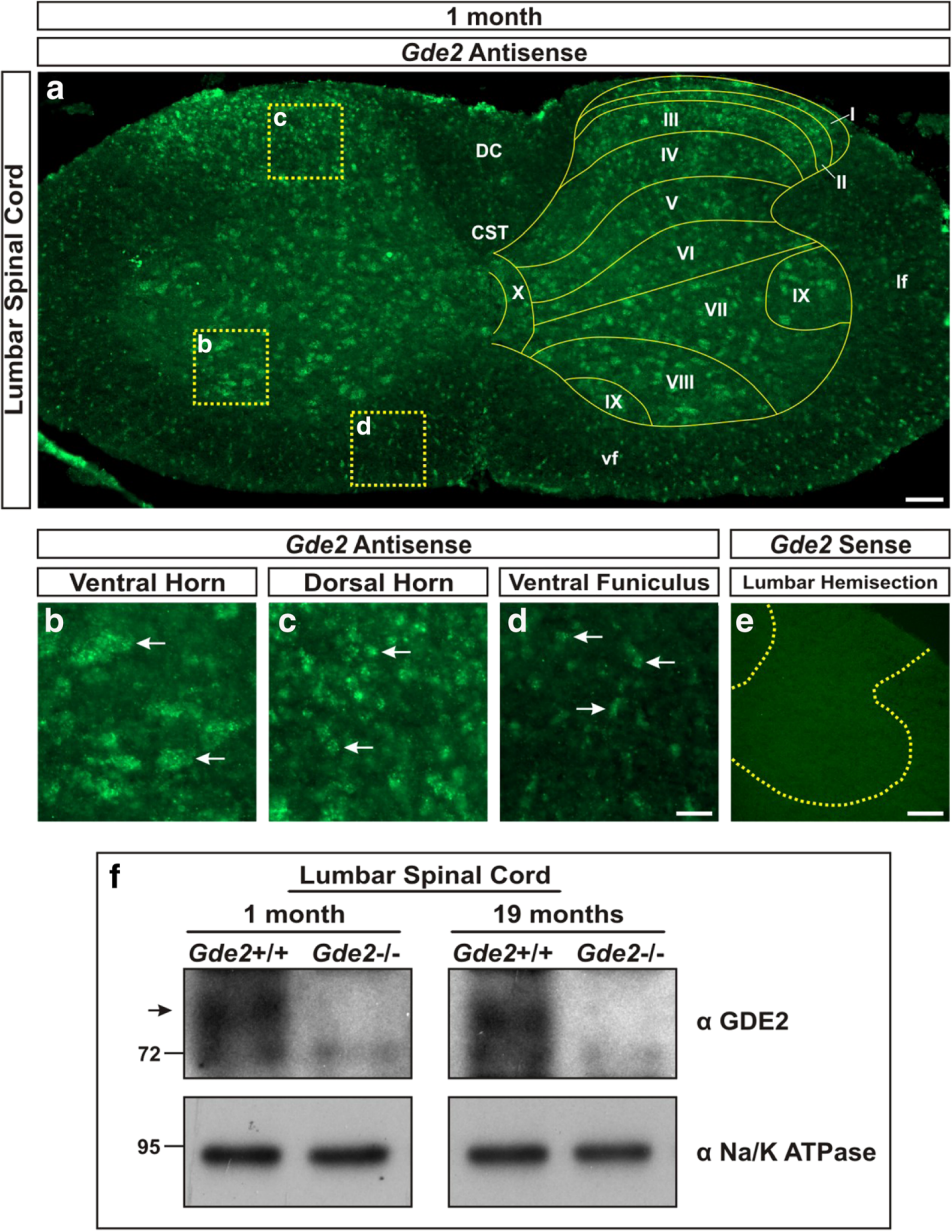
*Gde2* mRNA and protein are expressed in the postnatal spinal cord. **a** Paneled epifluorescent image of fluorescent in-situ hybridization (FISH) using a *Gde2* antisense probe showing broad expression in all spinal laminae (labeled I to X), as well as the corticospinal tract (CST), dorsal columns (DC), lateral funiculus (lf), and ventral funiculus (vf). Scale bar = 100 μm. **b**–**d** Magnified regions indicated in panel A showing *Gde2* expression in large diameter motor neurons (B), small diameter neurons (C), and glia (D). *Arrows* mark examples. Scale bar = 35 μm. **e** *Gde2* sense probe shows no signal in the lumbar spinal cord. Dashed *yellow* lines = boundary between *grey* and *white* matter. Scale bar = 150 μm. **f** Western blot visualizing GDE2 protein (80kD band) in WT and not *Gde2* KOs. *n* = 3

To assess postnatal expression of *Gde2*, we performed fluorescent in-situ hybridization (FISH) on lumbar spinal cord sections using antisense and sense probes directed against the 3′ untranslated region (UTR) of *Gde2*. In 1 month old *Gde2* ^+/+^ mice, *Gde2* mRNA expression is widespread throughout the spinal cord. We detect *Gde2* transcript in all spinal laminae, lateral and ventral funiculi, and the dorsal columns (Fig. 1a). This cellular distribution, particularly in the white matter, implies that postnatal *Gde2* expression is not confined to neuronal subtypes. We observe clear *Gde2* expression in large diameter neurons in the ventral horn (Fig. 1b), small diameter neurons in the dorsal horn (Fig. 1c), and glial soma in the white matter (Fig. 1d). Similar analysis using the corresponding sense probe showed no signal in adjacent sections confirming specificity of the antisense probe (Fig. 1e). This expression pattern is consistent with published cell-type expression databases documenting broad *Gde2* expression in the postnatal CNS [27, 28].

To confirm that GDE2 protein is produced postnatally, we performed western blots of 1 month and 19 month lumbar spinal cord lysates using an antibody generated against GDE2. *Gde2* ^+/+^ lysates show a strong band at the predicted size that is absent in *Gde2* ^−/−^ littermates (Fig. 1f). Taken together, these observations indicate that GDE2 expression continues throughout life, suggesting ongoing roles for GDE2 in the adult nervous system.

### *Gde2* null animals present degenerative pathology

**Fig. 2:**
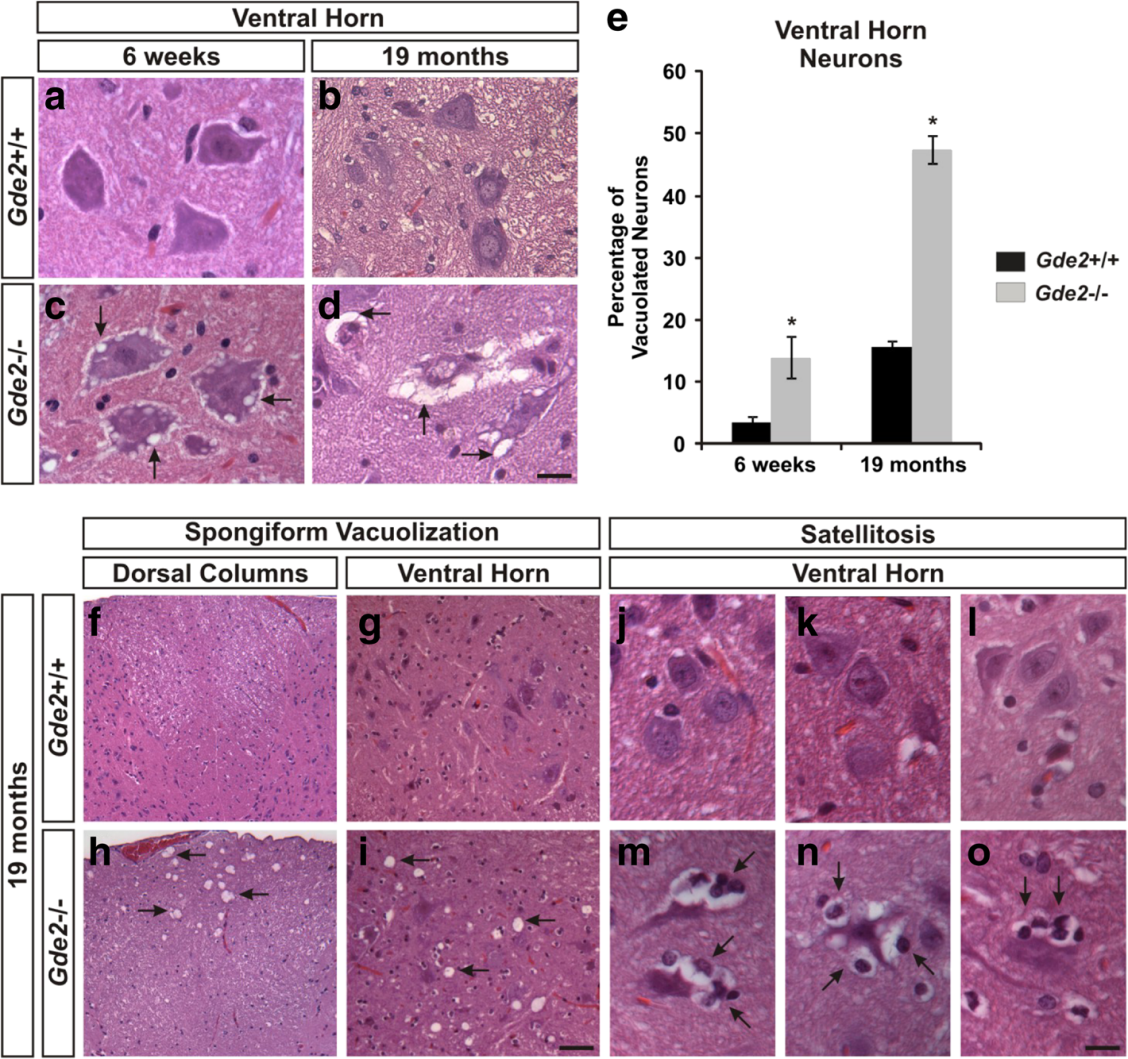
*Gde2* null animals develop vacuolization and satellitosis. **a**–**d** H&E staining of transverse sections of lumbar spinal cord. *Arrows* in **c**, **d** show progressive intracellular vacuolization. Scale bar = 20 μm. **e** Quantification of increased neuronal vacuolization in large diameter ventral horn neurons of the *Gde2* KO. Graph represents mean ± SEM. 6 weeks *p = 0.022, 19 months *p = <0.001, Student’s t test, *n* = 3. **f**–**i** *Arrows* mark large spongiform vacuoles in the dorsal columns and ventral horn. Scale bar = 60 μm. **j**–**o** *Arrows* mark satellitosis of ventral horn neurons of the *Gde2* KO. Scale bar = 20 μm. *n* = 3 WT, 4 KO

To investigate the postnatal roles of GDE2, we analyzed transverse sections of *Gde2* ^+/+^ and *Gde2* ^−/−^ lumbar spinal cords at 6 weeks and 19 months, two time points which allow the comparison between early and late stage pathology. Using Hematoxylin & Eosin (H&E) stains to visualize cellular and interstitial morphology, we find neurons in the *Gde2* ^−/−^ spinal cord are filled with intracellular vacuoles that are not apparent in wild-type (WT) animals (Fig. 2a–d). At 19 months, *Gde2* ^−/−^ motor neurons are heavily vacuolated, with shrunken and condensed somas, and have separated from their adjoining neuropil (Fig. 2d). We quantified the incidence of this intracellular vacuolization among neurons in the ventral horn. At 6 weeks, only 3.50 ± 1.09% of WT neurons contain vacuoles compared to 14.02 ± 3.39% in *Gde2* ^−/−^ mice. At 19 months, vacuolization among WT neurons climbs to 15.68 ± 1.03% and the incidence in the KO rises to 47.41 ± 2.28% (Fig. 2e). In addition, aged *Gde2* ^−/−^ animals exhibit prominent spongiform vacuoles in the extracellular space of the dorsal columns and ventral grey matter (Fig. 2f–i). We also observe instances of satellitosis, the surrounding of neurons by smaller nuclei that are presumptive macrophages in the processes of phagocytosis (Fig. 2j–o) [29]. These phenotypes point to a pronounced, progressive neuronal degeneration in the absence of GDE2.

### *Gde2* deletion causes astrocytosis and microgliosis

**Fig. 3:**
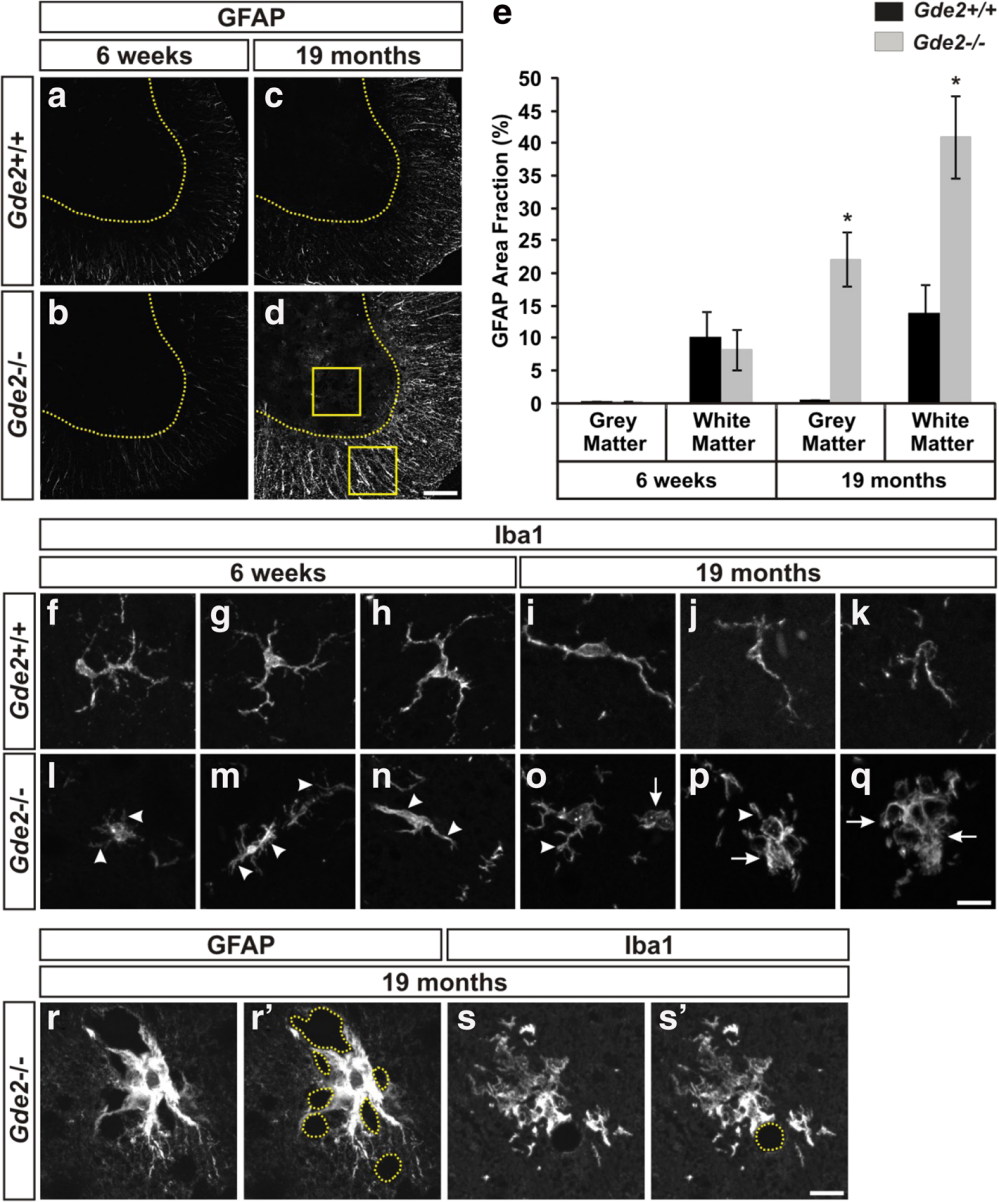
Astrocytosis and microgliosis in the *Gde2* KO. **a**–**d** Immunostaining for GFAP in WT (**a**, **c**) and *Gde2* KO (**b**, **d**). Dashed yellow lines = boundary between *grey* and *white* matter. Scale bar = 100 μm. **e** 19 month *Gde2* null *grey* and *white* matter have significantly elevated GFAP area fraction. Graph represents mean ± SEM. 19 month Grey matter *p = 0.002, 19 month White matter *p = 0.013, Student’s t test, *n* = 3. Example ROI positions are illustrated in panel **d**. **f**–**q** Confocal projections of Iba1^+^ microglia from the ventral horn of WT and *Gde2* KO ventral horns. *Arrowheads* indicate condensed microglial processes and arrows denote multicellular aggregations in the absence of *Gde2*. Scale bar = 15 μm. *n* = 3. **r**–**s’** Examples for GFAP^+^ astrocytes and Iba1^+^ microglial processes interacting with the spongiform vacuoles (*dashed yellow circles*) in the aged *Gde2* KO

Prompted by the pronounced satellitosis, we next analyzed *Gde2* ^−/−^ spinal cords for additional glial pathology. Gliosis refers to the inflammatory reaction of glial cells, mainly astrocytes and microglia, in response to injury or disease and is a strong indicator of degeneration. As astrocytes and microglia respond to disease conditions, they undergo distinct morphological transitions. Inflammatory astrocytes expand and enumerate their processes while microglia truncate their processes and form multicellular aggregates [30, 31]. We analyzed astrocyte morphology by staining for Glial Fibrillary Acidic Protein (GFAP). At 6 weeks, no appreciable differences are detected in the ventral horn of *Gde2* ^−/−^ mice (Fig. 3a and b); however, by 19 months, astrocytes in the *Gde2* ^−/−^ animals show intensified GFAP immunoreactivity and broadened processes (Fig. 3c and d). We quantified the differences in astrocyte morphology by calculating the area of all GFAP^+^ processes within a 150 μm^2^ region of interest (ROI) centered in either the ventral grey matter or white matter (Fig. 3d). The area of the GFAP^+^ processes divided by the area of the ROI yields the GFAP area fraction. At 6 weeks, the WT and KO values for GFAP area fraction were 0.22 ± 0.12% and 0.11 ± 0.08% in grey matter and 10.21 ± 3.97% and 8.29 ± 3.12% in white matter, respectively. At 19 months of age, the WT and KO values were 0.40 ± 0.22% and 22.26 ± 4.15% in grey matter and 13.86 ± 4.34% and 41.07 ± 6.40% in white matter, respectively (Fig. 3e). Resting Iba1^+^ microglia exhibit a very stereotyped morphology; they are evenly spaced with multiple thin branches extending from the soma (Fig. 3f–k). In contrast, microglia in the 6 week old *Gde2* KO spinal cord display shortened, condensed processes indicative of microglial activation (Fig. 3l–n). By 19 months, the reactive morphologies worsen and large microglial aggregates form in the ventral and lateral funiculi (Fig. 3o–q). In addition, we note that the inflammatory glia often associate with the spongiform abscesses in the grey and white matter (Fig. 3r–s’). Taken together, *Gde2* ablation produces prominent astro- and microgliosis, which suggests a progressive neuropathology in the *Gde2* KO spinal cord.

### *Gde2* KO neurons exhibit cytoskeletal accumulation and mislocalization

**Fig. 4:**
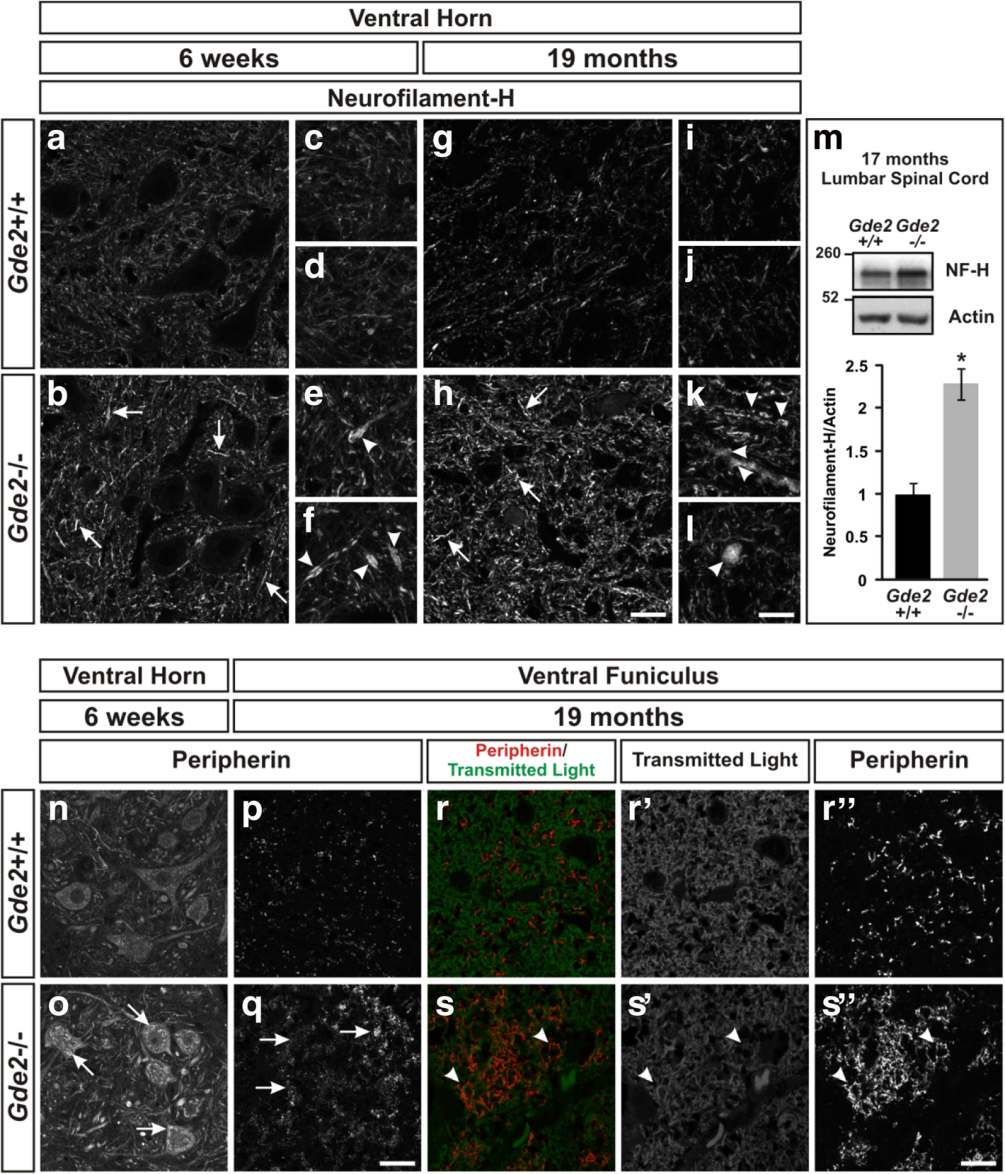
*Gde2* KO neurons show cytoskeletal dysregulation. **a**–**l** Neurofilament-H immunostaining in the ventral horn. *Arrows* indicate cytoskeletal accumulation in the absence of *Gde2*. *Arrowheads* mark spheroid deposits of Neurofilament-H within dilated neuronal processes. Scale bar **a**, **b**, **g**, **h** = 25 μm. Scale bar **c-f**, **i-l** = 15 μm. **m** Western blot quantifying increased NF-H protein. Graph represents mean ± SEM, *p = 0.004, Student’s t test, *n* = 3 WT, 4 KO. **n**–**q** *Gde2* null neurons accrue peripherin protein. Arrows indicate peripherin tendrils in the cell body at 6 weeks and axons at 19 months. Scale bar = 25 μm. **r**–**s”** Arrowheads denote axonal accumulation of peripherin enriched around the perimeter of the axon. Scale bar = 10 μm. *n* = 3

The neuronal pathology in the spinal cord motivated us to screen *Gde2* ^−/−^ animals for cytoskeletal defects prevalent in neurodegenerative disease. In neurons, one of the principal cytoskeletal components is Neurofilament, and of the three main neurofilament isoforms (NF-L, NF-M, NF-H), irregularities in NF-H expression have been most frequently associated with neurodegeneration [32–34]. In *Gde2* null animals at 6 weeks, we observe increased NF-H immunoreactivity within dilated neuronal processes (Fig. 4a–f). By 19 months, the magnitude of the cytoskeletal accumulation has risen in the ventral horn (Fig. 4g–l). To more accurately quantify the NF-H increase, we performed western blots of 17 month lumbar spinal cord lysates. We found a 2.25 ± 0.50 fold increase of NF-H protein expression in *Gde2* null spinal cords compared with WT (Fig. 4m). Peripherin is an intermediate filament expressed in motor, sensory, and sympathetic neurons and has also been reported to undergo degeneration dependent increases [35]. Unlike the bulk accumulation of NF-H, neurons lacking *Gde2* amassed peripherin protein within their somas at 6 weeks (Fig. 4n and o). At 19 months, we detect the majority of peripherin immunoreactivity in the ventral and lateral funiculi within axons. WT axons distribute peripherin in discrete bundles; *Gde2* ^−/−^ axons traffic peripherin to the interior perimeter of the axon (Fig. 4p–s”). Considering the importance of regulating cytoskeletal proteins for intracellular trafficking, the dysregulation of NF-H and peripherin pose a severe impediment to normal cellular function and likely reflect diminished neuronal health.

### *Gde2* KO neurons have accelerated lipofuscin deposition

**Fig. 5:**
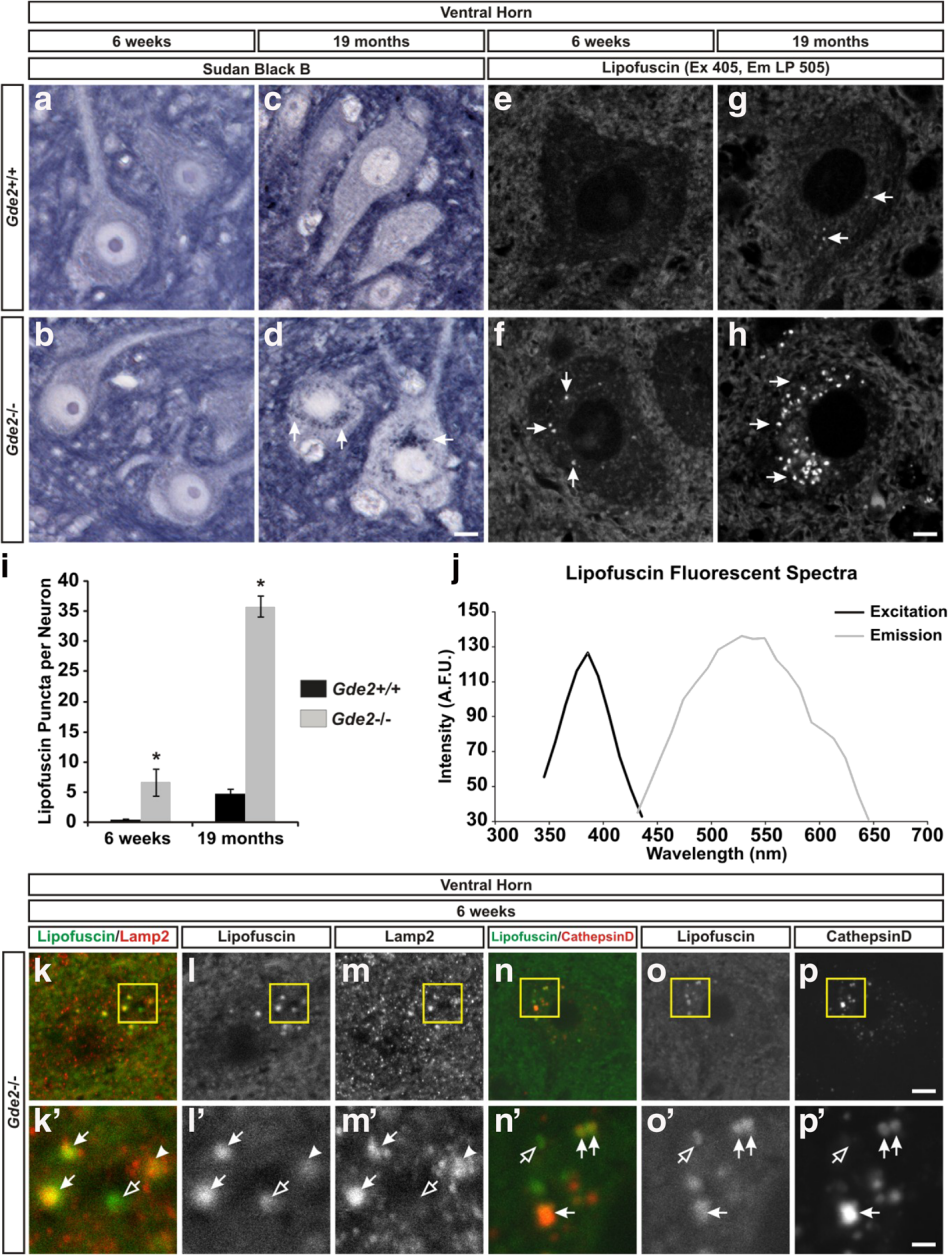
Lipid deposition in *Gde2* null neurons. **a**–**d** Sudan Black B darkly stains lipid deposits in motor neurons of the aged *Gde2* KO (*arrows*). Scale bar = 7 μm. *n* = 3. **e**–**h** Lipofuscin (*arrows*) is visualized as autofluorescent granules using ultraviolet excitation (405 nm) and a long pass emission (LP 505) filter. Scale bar = 10 μm, *n* = 3. **i** Quantification of elevated lipofuscin granules at 6 weeks *p = 0.028 and 19 months *p < 0.001, Student’s t test, *n* = 3. Graph represents mean ± SEM. **j** Lipofuscin granules have an ultraviolet excitation maxima centered at 390 nm, and a broad emission curve peaking at 530 nm. **k**, **l**, **m**, **n**, **o**, **p** Lipofuscin association with lysosomes labeled by Lamp2 or CathepsinD. Scale bar = 4.25 μm. **k’**, **l’**, **m’**, **n’**, **o’**, **p’** Magnification of boxed regions in panels **k-p**. Lipofuscin is fully or partially colocalized with (*filled arrows*), surrounded by (*arrowheads*), or devoid of (*open arrow*) lysosomal signal. Scale bar = 1.5 μm. *n* = 4

The degenerative accrual of NF-H and peripherin led us to investigate whether *Gde2* deletion promotes the pathologic accumulation of lipids as well as protein. Sudan Black B is a lipophilic dye that intercalates into lipid rich domains [36]. At 19 months, *Gde2* ^−/−^ neurons in the ventral horn have pronounced perinuclear deposits that strongly take up Sudan Black B that are not detectable in *Gde2* ^+/+^ neurons (Fig. 5a–d). These particles are characteristic of lipofuscin, an accumulation of lipids and undigested cellular debris, namely proteins, which collect as discrete cytosolic puncta during normal aging [37, 38]. Lipofuscin can be specifically visualized as autofluorescent granules with ultraviolet absorbance and broad emission in unstained tissue sections [36]. At 6 weeks of age, we detect an average of 6.68 ± 2.28 autofluorescent lipofuscin granules per neuron in the KO and 0.47 ± 0.18 granules in control neurons (Fig. 5e, f and i). At 19 months, numerous bright autofluorescent granules are evident in ventral horn neurons in *Gde2* KO animals compared with WT, 35.82 ± 1.69 versus 4.83 ± 0.84 granules, respectively (Fig. 5g–i). These deposits have a spectral excitation and emission profile consistent with lipofuscin (Fig. 5j). Lipofuscinogenesis is nucleated by the lysosome, wherein reactive hydroxyl radicals form aldehyde cross linkages between the undigested macromolecules [38]. If the autofluorescent granules seen in the *Gde2* KO are lipofuscin, we predicted that a subset should colocalize with lysosomal markers. Indeed, we find that some lipofuscin granules are fully or partially colocalized with Lamp2^+^ or CathepsinD^+^ lysosomes while others are juxtaposed, and some have no lysosomal signal (Fig. 5k–p’). Early accumulation of lipofuscin is an indicator of increased cellular stress and can presage neurodegeneration in disease conditions [39]. The advanced deposition of lipofuscin granules in *Gde2* ^−/−^ animals provides further evidence that GDE2 is essential for neuronal health.

### *Gde2* ablation causes progressive motor neuron loss

**Fig. 6:**
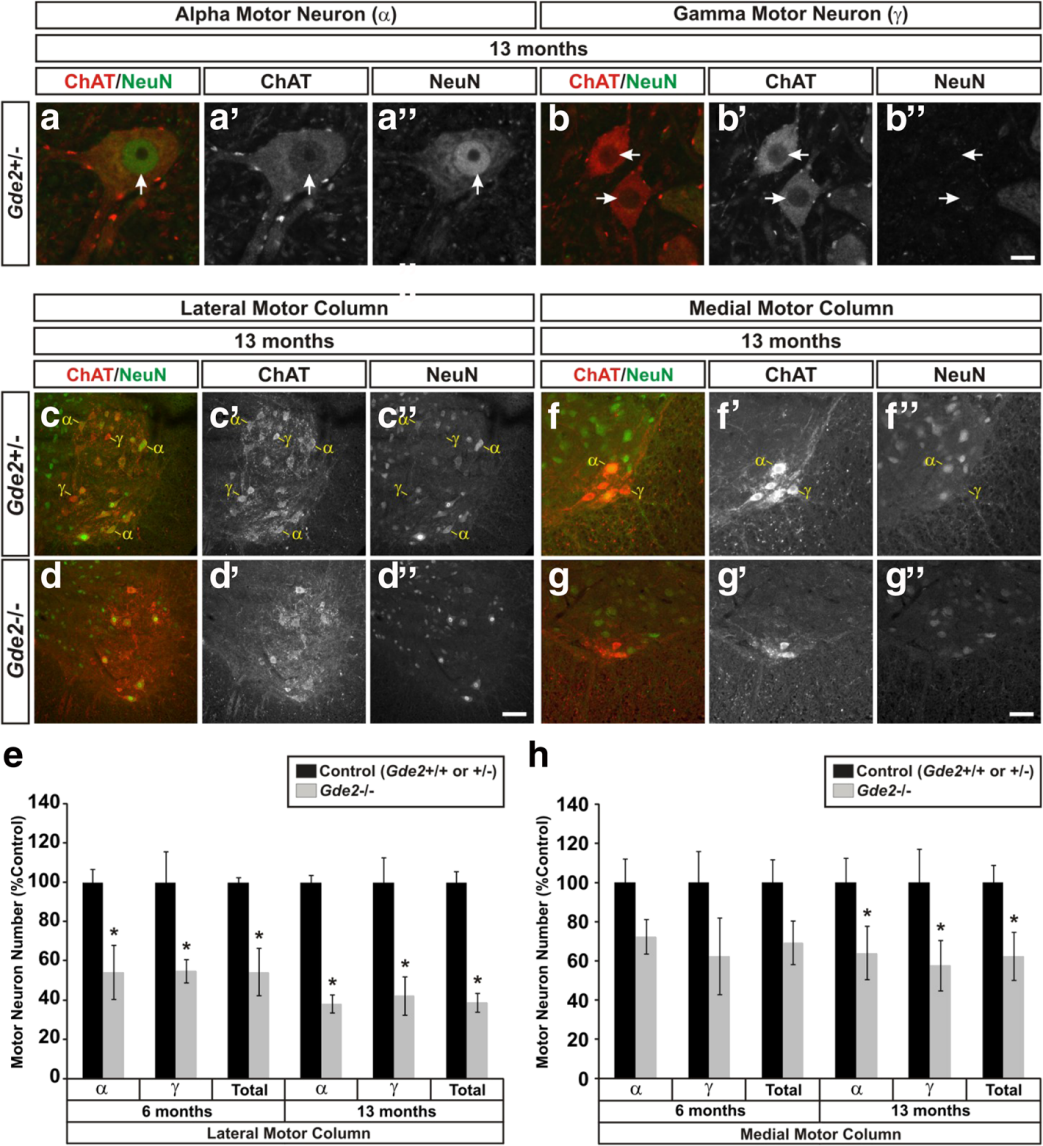
*Gde2* KO mice have progressive motor neuron loss. **a**–**b”** Example images of alpha (α) and gamma (γ) motor neurons. Alpha motor neurons are ChAT^+^/NeuN^+^ while gamma motor neurons are ChAT^+^/NeuN^−^. *Arrows* indicate position of the nucleus. Scale bar = 10 μm. **c**–**d”** Representative images of motor neurons in the LMC labeled with ChAT and NeuN at 13 months. Example α and γ motor neurons are indicated. Scale bar = 50 μm. **e** Quantification of LMC motor neuron loss. 6 months: α *p = 0.001, γ *p = 0.048, Total *p = 0.003, *n* = 3. 13 months: α *p < 0.001, γ *p = 0.046, Total *p = 0.001, *n* = 5. **f**–**g”** Images of α and γ motor neurons in the MMC at 13 months. **h** Graph quantifying MMC motor neuron decrements. 6 months: α *p* = 0.212, γ *p* = 0.151, Total *p* = 0.116, *n* = 3. 13 months: α *p = 0.002, γ *p < 0.001, Total *p = 0.002, *n* = 5. All graphs represent mean ± SEM, Student’s t test

The collective histology in the *Gde2* ^−/−^ spinal cord signifies a progressive neuropathology that worsens from 6 weeks to 19 months afflicting neurons in the ventral horn of the spinal cord. We next sought to understand if and how these pathologies impact neuronal survival. Though the pathology affects many neuronal subtypes in the ventral horn, we focused on motor neurons as their cell death is an integral component of many neurodegenerative diseases. We assessed the survival of alpha motor neurons (α) and gamma motor neurons (γ) in the lateral and medial motor columns (LMC and MMC) of the lumbar spinal cord. Alpha motor neurons innervate force generating skeletal muscle while gamma motor neurons innervate intrafusal muscle fibers to control proprioceptive sensory feedback [40]. Both types of motor neurons express Choline Acetyltransferase (ChAT) but only alpha motor neurons coexpress NeuN (Fig. 6a–b”) [41]. To understand how the progressive neuropathology that develops from 6 weeks to 19 months affects cell survival, we quantified motor neurons at 2 intermediate time points, 6 months and 13 months. In the LMC, which innervates limb muscles, we find a marked decrease in both alpha and gamma neurons (Fig. 6c–d”). At 6 months, we register a 45.84 ± 4.75% decrease in alpha motor neurons and a 45.07 ± 9.75% decrease in gamma motor neurons (Fig. 6e). By 13 months, these deficits grow to 61.91 ± 1.21% and 57.83 ± 1.87% for alpha and gamma, respectively (Fig. 6e). In the MMC, which innervates the axial muscles of the trunk, no decreases in alpha and gamma motor neurons are detected at 6 months; however, significant reductions are evident by 13 months of age with alpha motor neurons falling 35.83 ± 6.57% and gamma motor neurons dropping by 42.25 ± 3.03% in the *Gde2* KO (Fig. 6f–h). Thus, deletion of *Gde2* results in the progressive loss of spinal motor neurons as the animal ages.

### *Gde2* KO animals undergo peripheral restructuring without impaired peripheral nerve conduction

**Fig. 7:**
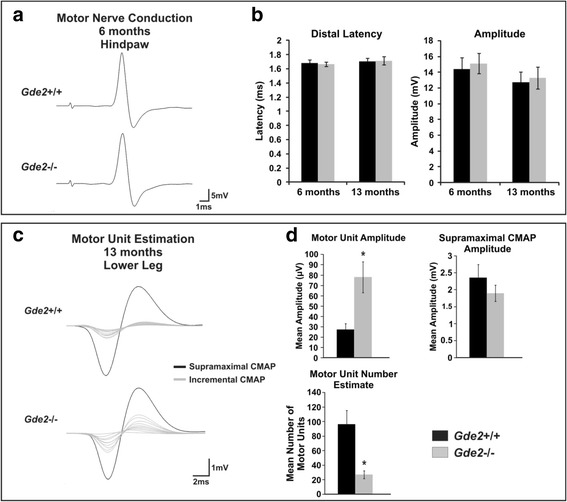
*Gde2* KO animals exhibit motor unit restructuring with equivalent peripheral conduction. **a** Example Compound Motor Action Potentials recorded from WT and *Gde2* KO hindpaws following supramaximal stimulation to the sciatic nerve. **b** Graphs measuring Distal Latency: 6 months *p* = 0.413, 13 months *p* = 0.457; and Amplitude: 6 months *p* = 0.356, 13 months *p* = 0.388, *n* = 10. **c** Motor Unit field recordings from lower hindlimb following incremental (*grey traces*) and supramaximal (*black traces*) stimulation of the sciatic nerve. **d** Graphs quantifying mean Motor Unit Amplitude *p = 0.002, Supramaximal CMAP Amplitude *p* = 0.144, and Motor Unit Number Estimate *p = 0.026, *n* = 5. All graphs represent mean ± SEM, Student’s t test

To determine if any physiological consequences result from the motor neuron deficits in the *Gde2* KO, we carried out a longitudinal electrophysiological study of peripheral nerve function in a cohort of 10 WT and 10 *Gde2* KO mice. Considering the significant motor neuron loss observed at 6 months and 13 months, we performed Motor Nerve Conduction Studies (MNCS) at these time points to screen for defects in peripheral nerve physiology. MNCS record a Compound Motor Action Potential (CMAP) from the plantar foot muscles after stimulating the sciatic nerve (Fig. 7a). The latency and amplitude of the CMAP convey the efficiency of action potential conduction and strength of the neuromuscular connection, respectively [42]. At 6 and 13 months, *Gde2* KO animals displayed no changes in CMAP waveforms (Fig. 7b), indicating preserved peripheral nerve function in the absence of *Gde2*.

We next measured the size of the individual motor units, defined as a motor axon and all the muscle fibers it innervates, using Motor Unit Number Estimation (MUNE). MUNE records from all the muscles of the lower hindlimb and utilizes sequential increases in sub-maximal stimulation to reveal individual CMAPs in a quantal manner, each representing one motor unit. After recording the amplitude of several motor units and dividing their average into the supramaximal CMAP, the number of motor units in the recording field can be estimated [43, 44]. We recorded the first ten stably detectable CMAPs from 13 month WT and *Gde2* nulls (Fig. 7c). The average motor unit amplitude increases from 0.0274 ± 0.005 mV in the WT to 0.0781 ± 0.015 mV in *Gde2* ^−/−^ animals (Fig. 7d). There is no change in the supramaximal CMAP; consequently, the MUNE values for WT and *Gde2* nulls are 96.48 ± 19 and 27.14 ± 5.23 motor units respectively (Fig. 7d). The decrease in motor units in *Gde2* ^*−/−*^ animals is consistent with peripheral restructuring observed during long-term progressive motor neuron loss, which involves reinnervation of denervated muscle fibers by sprouting collaterals [45].

### *Gde2* KO animals have impaired motor performance

**Fig. 8:**
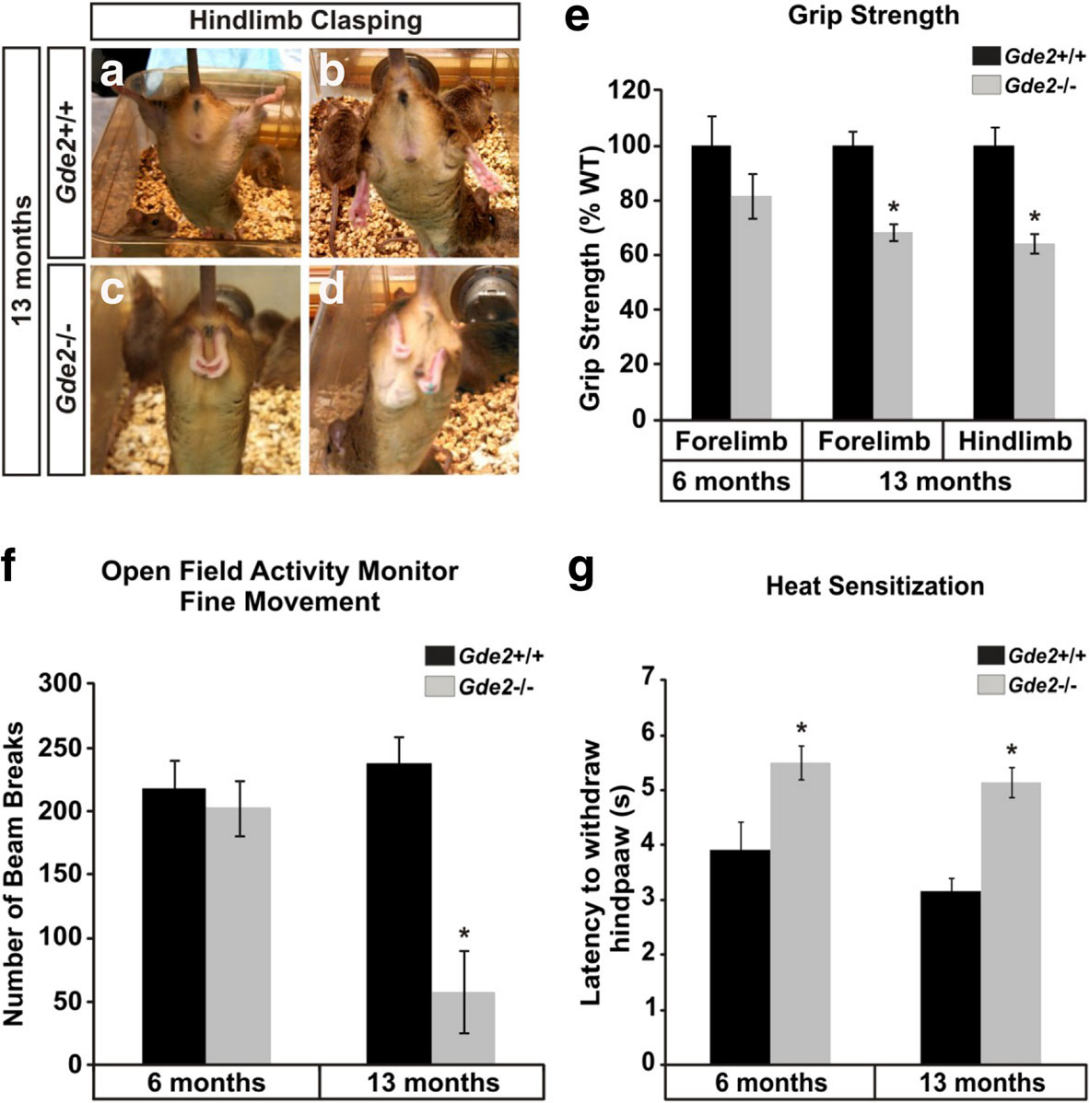
*Gde2* ablation causes progressive motor degeneration. **a**–**d** Aged *Gde2* KO animals display hindlimb clasping. **e** Grip strength shows significant interaction with genotype and animal age (F_1,39_ = 9.016, *p = 0.008) indicating progressive deterioration of *Gde2* KO motor performance. **f** Fine movement in the Open Field Activity Monitor reveals significant interaction with genotype (F_1,39_ = 4.45, *p = 0.049). **g** Heat sensitization shows significant increases at 6 months and 13 months in *Gde*2 KOs (F_1,39_ = 19,749, *p < 0.001, *n* = 10). Graphs represent mean ± SEM, 2-way repeated measures ANOVA, *n* = 10

Considering the neuronal loss, peripheral restructuring and histopathology of animals lacking GDE2, we measured the motor performance of our in-vivo cohort at 6 months and 13 months. When elevated by the tail, WT mice extend their hindlimbs away from their abdomen (Fig. 8a and b); however, the aged *Gde2* null mice display intermittent clasping of their hindlimbs consistent with motor neuropathy (Fig. 8c and d) [46]. Grip strength, the force that mice exert on a grid with their paws when lifted away [47], showed no difference at 6 months (Fig. 8e). However at 13 months, we observed a 31.52 ± 3.11% and 35.44 ± 3.63% decrease in forelimb and hindlimb grip strength, respectively (Fig. 8e). As with grip strength, we saw no changes in motor activity in an open field chamber [48] at 6 months but found a 73.47 ± 14.95% decrease in fine movement at 13 months (Fig. 8f). Lastly, we measured heat sensitization, the animal’s latency to withdraw its hindpaw from a noxious radiant heat source [48]. Successful completion of this task requires a functional sensorimotor reflex circuit, composed of a primary sensory neuron signaling to a motor neuron which in turn signals to the limb muscles. *Gde2* KO mice have an increased latency to withdraw their paw (5.51 ± 0.31 s versus 3.91 ± 0.52 s for the WT) as early as 6 months, and comparable delays are seen at 13 months of age (Fig. 8g), indicating deficits within the spinal sensorimotor reflex circuit. Considering the *Gde2* KO’s impaired heat sensitization, a task requiring sensory and motor function, we assayed primary sensory neurons of the dorsal root gangion (DRG) for evidence of pathology. We find that lumbar DRG neurons in the *Gde2* KO display analogous pathology to motor neurons, including vacuolization, lipid accumulation, and cytoskeletal dysregulation. As with efferent motor nerve conduction (Fig. 7b), we saw no changes in afferent peripheral sensory nerve conduction. (Additional file 1: Figure S1). These collective observations suggest that loss of GDE2 results in progressive erosion of sensorimotor function in the adult animal.

### GDE2 is required postnatally for neuronal survival

GDE2 is necessary during embryogenesis for promoting the differentiation of late-born alpha motor neurons within the limb-innervating LMC [8]. Aged *Gde2* KO animals show losses in LMC alpha motor neurons beyond the embryonic decrement and also exhibit reductions in gamma motor neurons and MMC motor neurons, two populations whose development is not impacted by *Gde2* deletion (Fig. 6e and h) [8]. These observations suggest that the degenerative loss of motor neurons in adult *Gde2* ^−/−^ animals is not a latent effect of improper embryonic development. To test this hypothesis, we utilized Cre-lox approaches to ablate *Gde2* after motor neuron differentiation, which is normally complete by E11.5. Specifically, a floxed conditional allele of *Gde2* (*Gde2* ^lox/-^) was used in concert with an inducible Cre^ER^ driven by the ubiquitously expressed *ROSA* promoter (*ROSA*:Cre^ER^) [49], and 4-hydroxytamoxifen (4-OHT) was delivered to pregnant dams at E17.5.

**Fig. 9:**
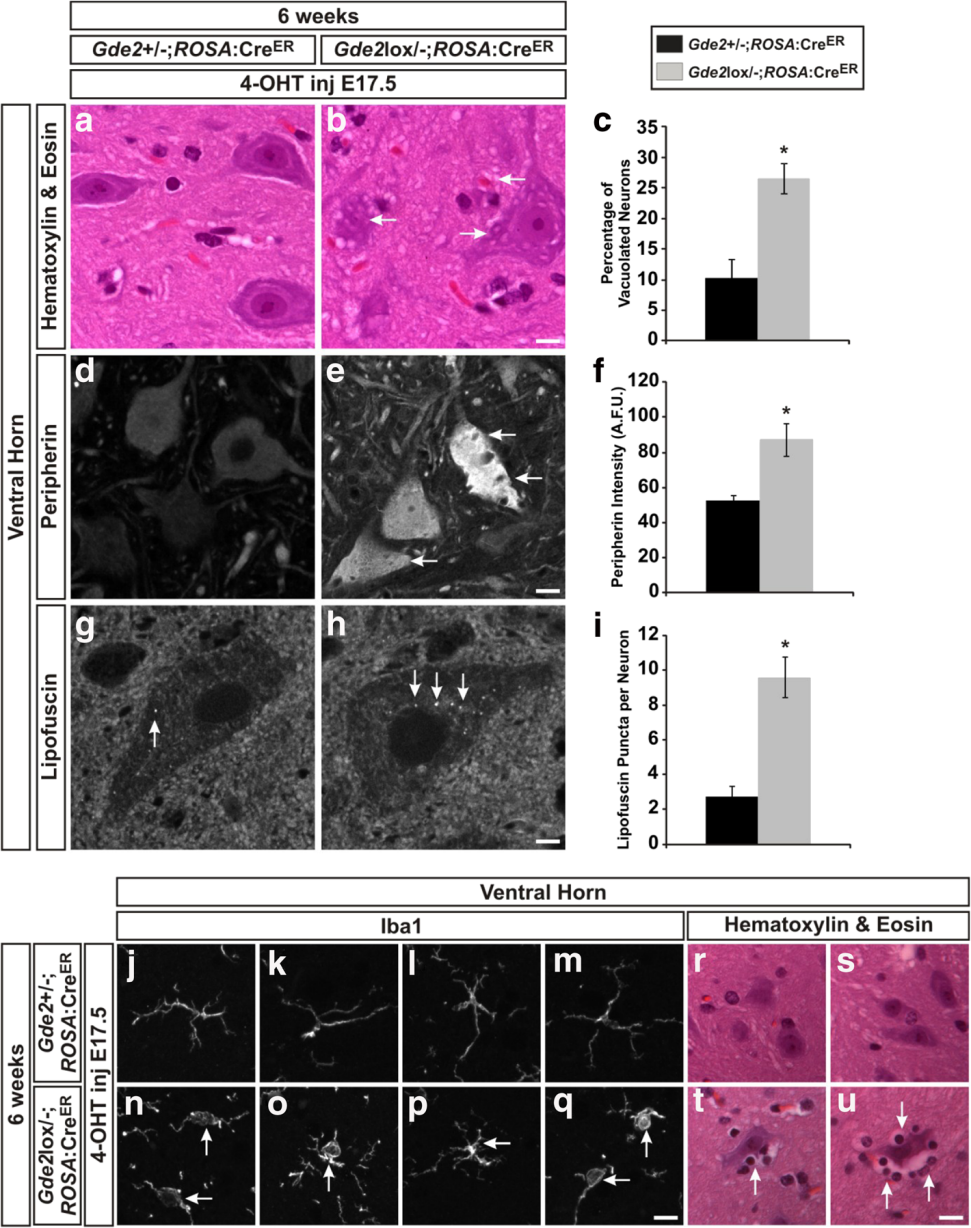
*Gde2* is required postnatally to prevent neurodegeneration. **a**, **b** H&E staining shows increased vacuolization in *Gde2* ^lox/-^;*ROSA*:Cre^ER^ motor neurons (*arrows*). Scale bar = 10 μm. **c** Quantification of increased vacuolization in *Gde2* ^lox/-^;*ROSA*:Cre^ER^ mice. *p = 0.007, *n* = 3. **d**, **e** Peripherin immunoreactivity is increased in motor neurons of *Gde2* ^lox/-^;*ROSA*:Cre^ER^ animal (*arrows*). Scale bar = 10 μm. **f** Graph quantifying increased peripherin signal in artificial fluorescence units (A.F.U). *p = 0.011, *n* = 3. **g**, **h** Enhanced lipofuscin accumulation is seen with the postnatal ablation of *Gde2* (*arrows*). Scale bar = 5 μm. **i** Quantification of lipofuscin granules per neuron. *p = 0.002, *n* = 3. Graphs represent mean ± SEM, Student’s t test. **j**–**q** Conditional ablation of *Gde2* induces aberrant microglial morphology (*arrows*), visualized by Iba1 staining. Scale bar = 10 μm. **r**–**u** *Gde2* ^lox/-^;*ROSA*:Cre^ER^ exhibit satellitosis (*arrows*) comparable to the constitutive *Gde2* KO. Scale bar = 20 μm. See also Additional file 1: Figure S2

We aged *Gde2* ^lox/-^;*ROSA*:Cre^ER^ and *Gde2* ^+/−^;*ROSA*:Cre^ER^ littermate controls to 6 weeks and first verified the efficient deletion of GDE2 at the functional and genetic level. Confirming the efficacy of temporal ablation after developmental neurogenesis is complete, the developmental reduction of LMC alpha motor neurons normally observed at 6 weeks in constitutive *Gde2* nulls is not present in *Gde2* ^lox/-^;*ROSA*:Cre^ER^ animals. Further, we verified the efficient ablation of the *Gde2* conditional allele using a competitive PCR strategy [8, 50] that shows near complete removal of the *Gde2* lox allele following 4-OHT administration (Additional file 1: Figure S2). We then analyzed these mice for the earliest signs of neurodegeneration exhibited by constitutive *Gde2* nulls at this time point, i.e. vacuolization, increased cytoskeletal protein, lipofuscin, and microgliosis. H&E staining revealed increased vacuolization in *Gde2* ^lox/-^;*ROSA*:Cre^ER^ motor neurons that is not seen in *Gde2* ^+/−^;*ROSA*:Cre^ER^ littermates (Fig. 9a and b). The incidence of vacuolization among ventral horn neurons rose from 10.25 ± 3.10% in the *Gde2* ^+/−^;*ROSA*:Cre^ER^ mouse to 26.56 ± 2.47% in the *Gde2* ^lox/-^;*ROSA*:Cre^ER^ (Fig. 9c). We found that *Gde2* ^lox/-^;*ROSA*:Cre^ER^ animals showed a substantial increase in somal peripherin immunoreactivity compared to controls (Fig. 9d and e). Mean peripherin intensity levels increased from 52.85 ± 2.66 artificial fluorescence units (A.F.U) in *Gde2* ^+/−^;*ROSA*:Cre^ER^ neurons to 87.34 ± 9.12 A.F.U. in the *Gde2* ^lox/-^;*ROSA*:Cre^ER^ mouse (Fig. 9f). Similarly, the average number of lipofuscin granules per neuron increases from 2.75 ± 0.56 in *Gde2* ^+/−^;*ROSA*:Cre^ER^ mice to 9.62 ± 1.18 in *Gde2* ^lox/-^;*ROSA*:Cre^ER^ animals (Fig. 9g-i). The conditional ablation of *Gde2* in the postnatal animal also reproduces the aberrant microglial morphology seen in the constitutive KO at 6 weeks. *Gde2* ^lox/-^;*ROSA*:Cre^ER^ microglia have swollen soma and less elaborate arborizations (Fig. 9j-q). *Gde2* ^lox/-^;*ROSA*:Cre^ER^ also exhibit satellitosis of neurons within the ventral horn (Fig. 9r–u). Taken together, these data indicate GDE2’s function in preventing neurodegeneration is distinct from its role in regulating motor neuron differentiation.

### Release of GPI-anchored GDE2 substrates is impaired in the *SOD1*^G93A^ mouse model of ALS

Our findings indicated that postnatal GDE2 function is critical to prevent degeneration, and raise the possibility that impaired GPI-anchor cleavage mediates neurodegeneration. To discover GPI-anchored proteins regulated by GDE2, we conducted an unbiased proteomic screen using alpha-toxin from *Clostridium septicum* which specifically binds to GPI-anchors [23]. We prepared non-membrane CNS extracts from aged WT and *Gde2* KO mice and used alpha-toxin to pull down GPI-anchored proteins that were released from the membrane. Protein complexes that yielded more signal in the WT, indicative of impaired GPI-anchor cleavage in the *Gde2* KO, were then identified using mass spectrometry. This screen identified two members of the Glypican family of heparin sulfate proteoglycans, Glypican 6 and Glypican 4, and both of these GPI-anchored proteins have been reported as substrates of GDE2 [9, 13]. We hypothesized that if the production of non-membrane bound Glypican 4 and Glypican 6 via GPI-anchor cleavage was crucial to prevent degeneration, we should see diminished production of these proteins in mouse models that exhibit motor neuron degeneration. The *SOD1* ^G93A^ transgenic mouse models familial ALS and shows profound motor neuron degeneration [51]. Although less severe, degeneration in *Gde2* nulls has considerable phenotypic overlap with the *SOD1* ^G93A^ model, namely vacuolization, neurofilament inclusions, gliosis, and motor neuron death [52, 53].

**Fig. 10:**
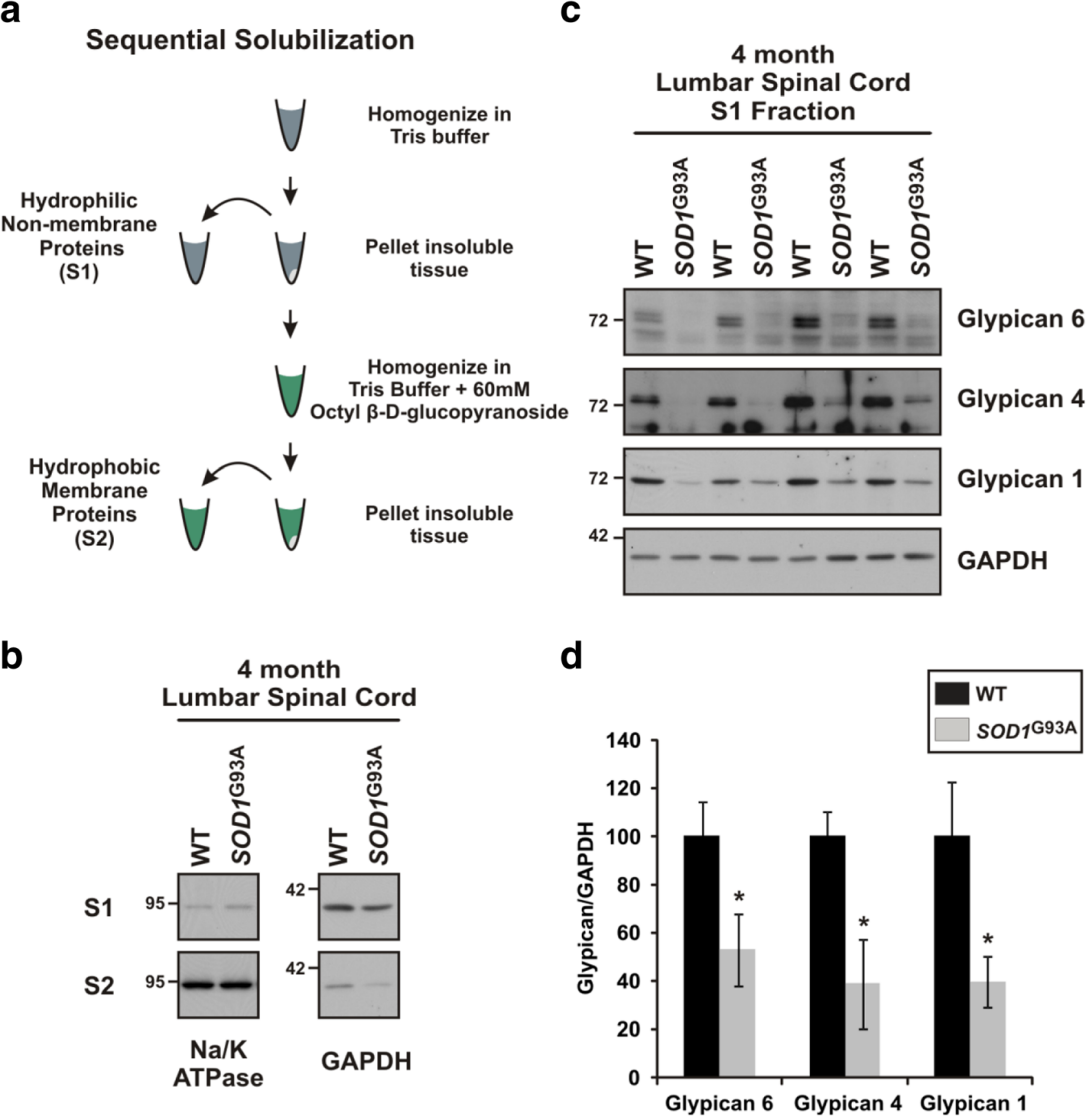
GDE2 substrates show impaired release in *SOD1* ^G93A^ mice. **a** Schematic of the sequential solubilization protocol used to separate hydrophilic non-membrane proteins (S1) from hydrophobic membrane proteins (S2). **b** Western blot demonstrating the successful enrichment of non-membrane protein (GAPDH) in the S1 fraction and membrane protein (Na/K ATPase) in the S2 fraction for WT and *SOD1* ^G93A^ transgenic mice. **c** Western blots visualizing the impaired production of non-membrane bound (S1) Glypican 6, Glypican 4, and Glypican 1 in the lumbar spinal cord of 4 month *SOD1* ^G93A^ mice. **d** Quantification of the reduced membrane release of Glypican 6, *p = 0.038; Glypican 4, *p = 0.016; and Glypican 1, *p = 0.030 relative to GAPDH. Graph represents mean ± SEM. Student’s t test, *n* = 4 WT, 4 *SOD1* ^G93A^

We aged *SOD1* ^G93A^ mice and their non-transgenic littermates (WT) to four months, an age with marked neurodegeneration, and separated protein samples from lumbar spinal cord extracts on the basis of hydrophobicity using sequential solubilization. We first produced an S1 fraction by solubilizing hydrophilic proteins in Tris buffer, and next an S2 fraction via solubilization of hydrophobic proteins in a buffer containing 60 mM Octyl β-D-glucopyranoside [54] (Fig. 10a). We confirmed the effective enrichment of non-membrane protein in the S1 fraction and membrane protein in S2 by performing western blots which show that GAPDH, a hydrophilic cytosolic protein, separates into S1, and Na/K ATPase a hydrophobic transmembrane protein separated into S2 (Fig. 10b). Since impaired membrane release of a GPI-anchored protein would reduce S1 levels, we compared Glypican 6 and Glypican 4 amounts in WT and *SOD1* ^G93A^ S1 fractions. We observe that *SOD1* ^G93A^ mice have reduced levels of Glypican 6 and Glypican 4 to 52.98 ± 14.87% and 38.82 ± 18.68% of control mice, respectively (Fig. 10c and d). In addition, we examined Glypican 1, a family member that has been connected with neurodegeneration in the context of Niemann-Pick Disease [17]. Similarly, we find that S1 Glypican 1 levels in the *SOD1* ^G93A^ spinal cord are reduced to 39.77 ± 10.73% of control littermates (Fig. 10c and d). These results indicate that the release of GPI-anchored Glypican 6, 4 and 1 is abated in the degenerating *SOD1* ^G93A^ mouse.

## Discussion

This report identifies a novel function for GDE2 in neuronal survival in the adult nervous system that is distinct from its function in embryogenesis. As early as 6 weeks, *Gde2* null animals exhibit pathologies analogous to human neurodegeneration including: intracellular vacuolization, microgliosis, accrual of cytoskeletal protein and deposition of lipofuscin. Later in life, *Gde2* KO animals show progressive reductions in motor neuron numbers, astrogliosis, and the appearance of spongiform vacuoles. Loss of motor neurons elicits peripheral sprouting evident by increases in motor unit size, culminating in deficits in motor behavior. Further, we show decreased membrane release of GPI-anchored GDE2 substrates belonging to the Glypican family in the *SOD1* ^G93A^ mouse model of familial ALS, suggesting that impaired GPI-anchor cleavage may mediate aspects of motor neuron degeneration.

### Postnatal versus embryonic functions of GDE2

GDE2 is expressed in spinal motor neurons during embryonic development and induces neighboring progenitors to differentiate into postmitotic motor neurons [6, 8]. We find that adult *Gde2* KO animals exhibit progressive spinal motor neuron degeneration. Though our analysis has focused on the lumbar spinal cord, comparable pathology is evident at cervical and thoracic levels. *Gde2* KO animals show an erosion of motor function over time, consistent with the age-dependent degeneration and loss of motor neurons in these animals. Our data argue against the possibility that loss of GDE2 during embryogenesis is responsible for the neurodegeneration observed in adult *Gde2* ^*−/−*^ animals. During neurogenesis, *Gde2* ablation disrupts the generation of late born alpha motor neurons within the LMC that innervate limb musculature; however, no changes are seen in MMC motor neurons which innervate axial muscles nor gamma motor neurons which regulate muscle stretch [8]. In contrast, cell loss in the adult *Gde2* KO encompasses alpha and gamma LMC neurons by 6 months and MMC neurons by 13 months. Thus, motor neuron degeneration in the adult *Gde2* KO involves motor neuron subtypes that are not dependent on GDE2 for their initial generation. In addition, selective ablation of GDE2 using an inducible Cre^ER^ system after motor neuron differentiation is complete still elicits neurodegenerative pathology. These collective observations suggest that GDE2 function in neuronal survival is distinct from its earlier role in controlling neurogenesis and is attributable to postnatal GDE2 expression in the nervous system. We note that GDE2 is also expressed in layer V cortical neurons [10]; it will be interesting to determine whether these neurons degenerate in the absence of GDE2 and if they also contribute to the motor deficiencies of *Gde2* KO animals.

Published profiling studies combined with our in-situ hybridization analyses show that postnatal GDE2 expression is not confined to neurons [27, 28]. While a cell-autonomous role for GDE2 in neurons is feasible, there is a growing body of literature implicating glia in neurodegeneration. Astrocytes, oligodendrocytes, and microglia have been identified as active components in the degeneration of neurons seen in mouse models of Amyotrophic Lateral Sclerosis (ALS) [55, 56] and Alzheimer’s Disease [57]. Moving forward it will be important to define the requisite cellular source of GDE2 for neuronal survival.

### GDE2 acts centrally to mediate neuronal survival

Neurodegeneration can initiate within the CNS or begin in the periphery and progress in a retrograde fashion towards cell bodies in the CNS, a process known as Wallerian-like degeneration [58]. Our study suggests that the degenerative pathology observed in *Gde2* KOs is not a consequence of Wallerian-like degeneration but is likely to initiate within the CNS itself. Analysis of quadriceps muscles in the *Gde2* KO does not reveal evidence of irregular neuromuscular junction (NMJ) morphology or denervated terminals at 19 months, a time when degenerative pathology is evident within neuronal cell bodies (Additional file 1: Figure S3). Moreover, we did not observe deficits in peripheral conduction times or action potential amplitude, indicating that peripheral axons, Schwann cells, and NMJs are relatively healthy. Further, mice lacking *Gde2* take longer to respond in a heat sensitization assay, a behavior that requires functional central and peripheral components. While this test cannot pinpoint which element of the sensorimotor reflex produces the latency, normal motor performance at 6 months and the integrity of peripheral nerve conduction suggest a defect in the central integration of the nociceptive signal rather than peripheral dysfunction. Taken together, these data imply that GDE2 functions centrally to maintain neuronal health and viability.

### Pathways regulated by GDE2 activity

Our developmental studies of GDE2 function identify GDE2 as a transmembrane enzyme that utilizes its extracellular enzymatic domain to cleave GPI-anchors that tether proteins to the membrane. Importantly, GDE2 and its family members GDE3 and GDE6 are the only known GPI-anchor cleaving enzymes in vertebrates that function at the cell surface, and accordingly, are key regulators of GPI-anchored protein activity at the plasma membrane [9]. This implies that GDE2 enzymatic regulation of specific GPI-anchored proteins is central to its function in neuronal survival. We conducted a proteomic screen to identify GPI-anchored substrates of GDE2 and found Glypican 4 and 6, both of which have been reported as GDE2 substrates [9, 13]. We then demonstrated that the non-membrane versions of these proteins, as well as Glypican 1, are reduced in *SOD1* ^G93A^ mice. These results support the hypothesis that the proper regulation of GPI-anchored proteins is crucial for neuronal survival. Moreover, they highlight Glypicans as potential components of pathways essential for neuronal viability whose dysfunction could lead to neurodegeneration. Investigating the role of GPI-anchored GDE substrates including Glypicans will be of major interest as we continue to define the molecular basis of neurodegeneration in *Gde2* KO animals as well as other models of degeneration.

How GDE2 mediates neuronal survival is a major outstanding question. Clues to the intracellular processes involved can be gleaned from the early degenerative phenotypes. *Gde2* KOs accumulate lipofuscin that is often associated or adjacent to Lamp2^+^ or CathepsinD^+^lysosomes. Lipofuscin is a complex mixture of oxidized protein, lipid degradation products, carbohydrates, and metals that is deposited during aging; however, the striking appearance of lipofuscin in *Gde2* null animals at 6 weeks is in line with a diseased neurodegenerative state rather than normal aging. Lipofuscin accumulation taxes lysosomal function and continually strains cellular clearance, [38] which can produce dire consequences for neuronal function and survival. Impaired cellular clearance may also generate the excess cytoskeletal protein in the *Gde2* KO. Disruptions in the cytoskeleton will impair intercellular membrane trafficking, potentially causing vacuolization. As this process begins to mount, first microglia and subsequently astrocytes become activated in response to neuronal stress, and as neurons die they are phagocytosed during satellitosis. Importantly, this model is compatible with the extended, progressive degeneration seen in the *Gde2* KO as chronic burdens on cellular clearance can slowly deteriorate neuronal health over time [38]. Future experiments will address the precise interrelation of the early *Gde2* null pathology to better understand which subcellular processes instigate the degeneration.

### GDE2 and disease

This report shows GDE2, a GPI-anchor cleaving enzyme previously identified as necessary for embryonic neuronal differentiation, is a novel survival factor for motor neurons in the postnatal spinal cord, and provides new insight into the fundamental mechanisms governing neuronal survival. Could GDE2 hypofunctionality be an unrecognized component in human disease? Although *Gde2* is expressed in humans [59], no disease pedigrees have been linked with mutations in *Gde2*. However, GDE2 could be functionally inhibited in disease conditions, producing pathology without registering mutations at the genomic level. GDE2 activity is highly sensitive to cellular redox states. High oxidative conditions in the endoplasmic reticulum oxidize cysteine residues within the GDE2 enzymatic domain and prevent GDE2 trafficking to the cell surface [60]. In addition, GDE2 activity at the cell surface can be inhibited by oxidation of cysteine residues within the intracellular N- and C-termini [7]. We speculate that increased ROS levels in disease conditions could lead to thiol-redox dependent inhibition of GDE2 trafficking and/or activity that would impinge on its function in neuronal survival. Importantly, elevation of reactive oxygen species (ROS) and oxidative stress are prevalent in many neurodegenerative diseases [61–64], broadening the potential for GDE2 inhibition in disease.

*Gde2* was recently identified in a network analysis that generated a broad “HSPome” of potential targets of Hereditary Spastic Paraplegia [65]. Interestingly, the slow rate of motor neuron degeneration in *Gde2* KOs is similar to HSP and certain forms of Spinal Muscular Atrophy [66, 67]. In addition, decreased expression of *Gde2* has been observed in laser captured motor neurons from sporadic ALS patients [68]. These observations reinforce the compelling notion that GDE2 dysfunction may underlie aspects of human neurodegenerative disease. Moving forward, verifying GDE2 hypofunctionality in human disease will be of paramount importance, and elucidating the mechanisms by which GDE2 regulates neuronal survival will be crucial to develop strategies to mitigate neurodegeneration.

## Conclusion

This report identifies GDE2 as a novel survival factor for spinal neurons in the adult nervous system. *Gde2* knock-out animals exhibit a progressive neurodegeneration marked by pathologies mirroring human disease. Conditional ablation of *Gde2* demonstrates that GDE2 functions postnatally to ensure neuronal health and survival. In addition, we find that the production of membrane released Glypicans, GPI-anchored substrates of GDE2, is reduced in the *SOD1* ^G93A^ mouse model. Our findings implicate diminished GDE2 function as a novel component in the etiology of several diverse neurodegenerative pathologies, and raise the possibility that improper regulation of GPI-anchored GDE2 substrates is mechanistically linked to neurodegeneration.

## Additional files

Additional file 1: Figure S1. Sensory neurons exhibit neurodegenerative pathology without impaired nerve conduction in the absence of *Gde2.* This figure illustrates the presence of neuropathology in the primary sensory neurons of the *Gde2* KO, including vacuolization, lipid accrual, and cytoskeletal accumulation; and the maintenance of peripheral sensory nerve conduction. **Figure S2**-related to Fig. 9. Conditional ablation of *Gde2* in the postnatal spinal cord prevents the developmental loss of motor neurons. This figure confirms the effective conditional ablation of *Gde2* following neurogenesis. In the constitutive KO, GDE2’s absence during embryonic neurogenesis causes a reduction of alpha motor neurons in the lateral motor column; however, in the Gde2^lox/-;^ *ROSA*:Cre^ER^ animals, this loss is avoided by injecting 4-OHT at E17.5. Further, competitive PCR analysis shows a near complete deletion of the conditional *Gde2* allele following 4-OHT delivery. **Figure S3.** *Gde2* deletion does not perturb neuromuscular junction morphology. This figure uses wholemount immunohistochemistry to assess the integrity of the neuromuscular junction (NMJ) in aged *Gde2* KO hindlimb muscle. At 19 months, no discernible pathology is present in the *Gde2* KO NMJ. (DOCX 6586 kb)

## Abbreviations

4-OHT: 4-Hydroxytamoxifen
AD: Alzheimer’s Disease
AFU: Artificial fluorescence units
ALS: Amyotrophic Lateral Sclerosis
ChAT: Choline Acetyltransferase
CMAP: Compound Motor Action Potential
DRG: Dorsal Root Ganglion
FISH: Fluorescent in-situ hybridization
GDE2: Glycerophosphodiester phosphodiesterase 2
GDPD: Glycerophosphodiester phosphodiesterase
GPI: Glycosylphosphatidylinositol
H&E: Hematoxylin and Eosin
KO: Knock out
LMC: Lateral motor column
MMC: Medial motor column
MN: Motor neuron
MNCS: Motor Nerve Conduction Study
MUNE: Motor Unit Number Estimation
NF-H: Neurofilamant-H
NMJ: Neuromuscular junction
RECK: REversion-inducing Cysteine-rich protein with Kazal motifs
ROS: Reactive oxygen species
SNAP: Sensory Nerve Action Potential
WT: Wild type

## References

[1] Gould E (2007). How widespread is adult neurogenesis in mammals?. Nat Rev Neurosci.

[2] Bjornsson CS, Apostolopoulou M, Tian Y, Temple S (2015). It takes a village: constructing the neurogenic niche. Dev Cell.

[3] Corda D, Mosca MG, Ohshima N, Grauso L, Yanaka N, Mariggiò S (2014). The emerging physiological roles of the glycerophosphodiesterase family. FEBS J.

[4] Yanaka N (2007). Mammalian glycerophosphodiester phosphodiesterases. Biosci Biotechnol Biochem.

[5] Corda D, Kudo T, Zizza P, Iurisci C, Kawai E, Kato N, Yanaka N, Mariggiò S (2009). The developmentally regulated osteoblast phosphodiesterase GDE3 is glycerophosphoinositol-specific and modulates cell growth. J Biol Chem.

[6] Rao M, Sockanathan S (2005). Transmembrane protein GDE2 induces motor neuron differentiation in vivo. Science.

[7] Yan Y, Sabharwal P, Rao M, Sockanathan S (2009). The antioxidant enzyme Prdx1 controls neuronal differentiation by thiol-redox-dependent activation of GDE2. Cell.

[8] Sabharwal P, Lee C, Park S, Rao M, Sockanathan S (2011). GDE2 regulates subtype-specific motor neuron generation through inhibition of Notch signaling. Neuron.

[9] Park S, Lee C, Sabharwal P, Zhang M, Meyers CLF, Sockanathan S (2013). GDE2 Promotes Neurogenesis by Glycosylphosphatidylinositol-Anchor Cleavage of RECK. Science.

[10] Rodriguez M, Choi J, Park S, Sockanathan S (2012). Gde2 regulates cortical neuronal identity by controlling the timing of cortical progenitor differentiation. Development.

[11] Chatterjee S, Mayor S (2001). The GPI-anchor and protein sorting. Cell Mol Life Sci.

[12] The UniProt Consortium (2014). UniProt: a hub for protein information. Nucleic Acids Res.

[13] Matas-Rico E, van Veen M, Leyton-Puig D, van den Berg J, Koster J, Kedziora KM, Molenaar B, Weerts MJA, de Rink I, Medema RH, Giepmans BNG, Perrakis A, Jalink K, Versteeg R, Moolenaar WH (2016). Glycerophosphodiesterase GDE2 promotes neuroblastoma differentiation through glypican release and is a marker of clinical outcome. Cancer Cell.

[14] Radford HE, Mallucci GR (2010). The role of GPI-anchored PrP C in mediating the neurotoxic effect of scrapie prions in neurons. Curr Issues Mol Biol.

[15] Yang LB, Li R, Meri S, Rogers J, Shen Y (2000). Deficiency of complement defense protein CD59 may contribute to neurodegeneration in Alzheimer’s disease. J Neurosci.

[16] Watanabe N (2004). Glypican-1 as an A binding HSPG in the human brain: Its localization in DIG domains and possible roles in the pathogenesis of Alzheimer’s disease. FASEB J.

[17] Mani K, Cheng F, Fransson L-A (2006). Defective nitric oxide-dependent, deaminative cleavage of glypican-1 heparan sulfate in Niemann-Pick C1 fibroblasts. Glycobiology.

[18] Glas M, Popp B, Angele B, Koedel U, Chahli C, Schmalix WA, Anneser JM, Pfister HW, Lorenzl S (2007). A role for the urokinase-type plasminogen activator system in amyotrophic lateral sclerosis. Exp Neurol.

[19] Lagier-Tourenne C, Polymenidou M, Hutt KR, Vu AQ, Baughn M, Huelga SC, Clutario KM, Ling S-C, Liang TY, Mazur C, Wancewicz E, Kim AS, Watt A, Freier S, Hicks GG, Donohue JP, Shiue L, Bennett CF, Ravits J, Cleveland DW, Yeo GW (2012). Divergent roles of ALS-linked proteins FUS/TLS and TDP-43 intersect in processing long pre-mRNAs. Nat Neurosci.

[20] Wong PC, Cai H, Borchelt DR, Price DL (2002). Genetically engineered mouse models of neurodegenerative diseases. Nat Neurosci.

[21] Rothstein JD (2009). Current hypotheses for the underlying biology of amyotrophic lateral sclerosis. Ann Neurol.

[22] Schaeren-Wiemers N, Gerfin-Moser A (1993). A single protocol to detect transcripts of various types and expression levels in neural tissue and cultured cells: in situ hybridization using digoxigenin-labelled cRNA probes. Histochemistry.

[23] Zhao P, Nairn AV, Hester S, Moremen KW, O’Regan RM, Oprea G, Wells L, Pierce M, Abbott KL (2012). Proteomic identification of glycosylphosphatidylinositol anchor-dependent membrane proteins elevated in breast carcinoma. J Biol Chem.

[24] Shevchenko A, Wilm M, Vorm O, Mann M (1996). Mass spectrometric sequencing of proteins silver-stained polyacrylamide gels. Anal Chem.

[25] Peng J, Gygi SP (2001). Proteomics: the move to mixtures. J Mass Spectrom.

[26] Eng JK, McCormack AL, Yates JR (1994). An approach to correlate tandem mass spectral data of peptides with amino acid sequences in a protein database. J Am Soc Mass Spectrom.

[27] Cahoy JD, Emery B, Kaushal A, Foo LC, Zamanian JL, Christopherson KS, Xing Y, Lubischer JL, Krieg PA, Krupenko SA, Thompson WJ, Barres BA (2008). A transcriptome database for astrocytes, neurons, and oligodendrocytes: a new resource for understanding brain development and function. J Neurosci.

[28] Zhang Y, Chen K, Sloan SA, Bennett ML, Scholze AR, O’Keeffe S, Phatnani HP, Guarnieri P, Caneda C, Ruderisch N, Deng S, Liddelow SA, Zhang C, Daneman R, Maniatis T, Barres BA, Wu JQ (2014). An RNA-sequencing transcriptome and splicing database of glia, neurons, and vascular cells of the cerebral cortex. J Neurosci.

[29] Garman RH (2011). Histology of the central nervous system. Toxicol Pathol.

[30] Kreutzberg GW (1996). Microglia: a sensor for pathological events in the CNS. Trends Neurosci.

[31] Barres BA (2008). The mystery and magic of glia: a perspective on their roles in health and disease. Neuron.

[32] Xiao S, McLean J, Robertson J (2006). Neuronal intermediate filaments and ALS: a new look at an old question. Biochim Biophys Acta.

[33] Gray E, Rice C, Nightingale H, Ginty M, Hares K, Kemp K, Cohen N, Love S, Scolding N, Wilkins A (2013). Accumulation of cortical hyperphosphorylated neurofilaments as a marker of neurodegeneration in multiple sclerosis. Mult Scler.

[34] Lalonde R, Strazielle C (2003). Neurobehavioral characteristics of mice with modified intermediate filament genes. Rev Neurosci.

[35] Bruijn LI, Miller TM, Cleveland DW (2004). Unraveling the mechanisms involved in motor neuron degeneration in ALS. Annu Rev Neurosci.

[36] Schnell SA, Staines WA, Wessendorf MW (1999). Reduction of lipofuscin-like autofluorescence in fluorescently labeled tissue. J Histochem Cytochem.

[37] Frank A, Christensen A (1968). Localization of acid phosphatase in lipofuscin granules and possible autophagic vacuoles in interstitial cells of the guinea pig testis. J Cell Biol.

[38] Gray DA, Woulfe J (2005). Lipofuscin and aging: a matter of toxic waste. Sci Aging Knowl Environ.

[39] Mink JW, Augustine EF, Adams HR, Marshall FJ, Kwon JM (2013). Classification and natural history of the neuronal ceroid lipofuscinoses. J Child Neurol..

[40] Kanning KC, Kaplan A, Henderson CE (2010). Motor neuron diversity in development and disease. Annu Rev Neurosci..

[41] Friese A, Kaltschmidt JA, Ladle DR, Sigrist M, Jessell TM, Arber S (2009). Gamma and alpha motor neurons distinguished by expression of transcription factor Err3. Proc Natl Acad Sci..

[42] Keswani SC, Jack C, Zhou C, Höke A (2006). Establishment of a rodent model of HIV-associated sensory neuropathy. J Neurosci..

[43] Shefner JM, Cudkowicz M, Brown RH (2006). Motor unit number estimation predicts disease onset and survival in a transgenic mouse model of amyotrophic lateral sclerosis. Muscle Nerve..

[44] Shefner JM (2009). Recent MUNE studies in animal models of motor neuron disease. Suppl Clin Neurophysiol..

[45] Daube JR (2006). Motor unit number estimates--from A to Z. J Neurol Sci..

[46] Filali M, Lalonde R, Rivest S (2011). Sensorimotor and cognitive functions in a SOD1(G37R) transgenic mouse model of amyotrophic lateral sclerosis. Behav Brain Res..

[47] Brooks SP, Dunnett SB (2009). Tests to assess motor phenotype in mice: a user’s guide. Nat Rev Neurosci..

[48] Le Bars D, Gozariu M, Cadden SW (2001). Animal models of nociception. Pharmacol Rev..

[49] Badea TC, Wang Y, Nathans J (2003). A noninvasive genetic/pharmacologic strategy for visualizing cell morphology and clonal relationships in the mouse. J Neurosci.

[50] Kim K, Jarry H, Knoke I, Seong JY, Leonhardt S, Wuttke W (1993). Competitive PCR for quantitation of gonadotropin-releasing hormone mRNA level in a single micropunch of the rat preoptic area. Mol Cell Endocrinol.

[51] Gurney ME, Pu H, Chiu AY, Dal Canto MC, Polchow CY, Alexander DD, Caliendo J, Hentati A, Kwon YW, Deng HX (1994). Motor neuron degeneration in mice that express a human Cu, Zn superoxide dismutase mutation. Science.

[52] Vinsant S, Mansfield C, Jimenez-Moreno R, Moore VDG, Yoshikawa M, Hampton TG, Prevette D, Caress J, Oppenheim RW, Milligan C (2013). Characterization of early pathogenesis in the SOD1G93A mouse model of ALS: Part II, results and discussion. Brain Behav.

[53] Vinsant S, Mansfield C, Jimenez-Moreno R, Del Gaizo Moore V, Yoshikawa M, Hampton TG, Prevette D, Caress J, Oppenheim RW, Milligan C (2013). Characterization of early pathogenesis in the SOD1(G93A) mouse model of ALS: part I, background and methods. Brain Behav.

[54] Hooper NM, Turner AJ (1988). Ectoenzymes of the kidney microvillar membrane. Differential solubilization by detergents can predict a glycosyl-phosphatidylinositol membrane anchor. Biochem J.

[55] Yamanaka K, Chun SJ, Boillee S, Fujimori-Tonou N, Yamashita H, Gutmann DH, Takahashi R, Misawa H, Cleveland DW (2008). Astrocytes as determinants of disease progression in inherited amyotrophic lateral sclerosis. Nat Neurosci.

[56] Lee Y, Morrison BM, Li Y, Lengacher S, Farah MH, Hoffman PN, Liu Y, Tsingalia A, Jin L, Zhang P-W, Pellerin L, Magistretti PJ, Rothstein JD (2012). Oligodendroglia metabolically support axons and contribute to neurodegeneration. Nature.

[57] Hong S, Beja-Glasser VF, Nfonoyim BM, Frouin A, Li S, Ramakrishnan S, Merry KM, Shi Q, Rosenthal A, Barres BA, Lemere CA, Selkoe DJ, Stevens B (2016). Complement and microglia mediate early synapse loss in Alzheimer mouse models. Science.

[58] Chaudhry V, Glass JD, Griffin JW (1992). Wallerian degeneration in peripheral nerve disease. Neurol Clin.

[59] Uhlén M, Fagerberg L, Hallström BM, Lindskog C, Oksvold P, Mardinoglu A, Sivertsson Å, Kampf C, Sjöstedt E, Asplund A, Olsson I, Edlund K, Lundberg E, Navani S, Szigyarto CA-K, Odeberg J, Djureinovic D, Takanen JO, Hober S, Alm T, Edqvist P-H, Berling H, Tegel H, Mulder J, Rockberg J, Nilsson P, Schwenk JM, Hamsten M, von Feilitzen K, Forsberg M (2015). Proteomics. Tissue-based map of the human proteome. Science.

[60] Yan Y, Wladyka C, Fujii J, Sockanathan S (2015). Prdx4 is a compartment-specific H2O2 sensor that regulates neurogenesis by controlling surface expression of GDE2. Nat Commun.

[61] Cobb CA, Cole MP (2015). Oxidative and nitrative stress in neurodegeneration. Neurobiol Dis.

[62] Simpson EP, Henry YK, Henkel JS, Smith RG, Appel SH (2004). Increased lipid peroxidation in sera of ALS patients: a potential biomarker of disease burden. Neurology.

[63] Markesbery WR, Lovell MA (1998). Four-hydroxynonenal, a product of lipid peroxidation, is increased in the brain in Alzheimer’s disease. Neurobiol Aging.

[64] Alam ZI, Daniel SE, Lees AJ, Marsden DC, Jenner P, Halliwell B (1997). A generalised increase in protein carbonyls in the brain in Parkinson’s but not incidental Lewy body disease. J Neurochem.

[65] Novarino G, Fenstermaker AG, Zaki MS, Hofree M, Silhavy JL, Heiberg AD, Abdellateef M, Rosti B, Scott E, Mansour L, Masri A, Kayserili H, Al-Aama JY, Abdel-Salam GM, Karminejad A, Kara M, Kara B, Bozorgmehri B, Ben-Omran T, Mojahedi F, Mahmoud IG, Bouslam N, Bouhouche A, Benomar A, Hanein S, Raymond L, Forlani S, Mascaro M, Selim L, Shehata N (2014). Exome sequencing links corticospinal motor neuron disease to common neurodegenerative disorders. Science.

[66] Zanoteli E, Maximino JR, Conti Reed U, Chadi G (2010). Spinal muscular atrophy: from animal model to clinical trial. Funct Neurol.

[67] Fink JK (2014). Hereditary spastic paraplegia: clinical principles and genetic advances. Semin Neurol.

[68] Rabin SJ, Kim JMH, Baughn M, Libby RT, Kim YJ, Fan Y, Libby RT, La Spada A, Stone B, Ravits J (2010). Sporadic ALS has compartment-specific aberrant exon splicing and altered cell-matrix adhesion biology. Hum Mol Genet.

