# Supplementary material for: GDE2 is essential for neuronal survival in the postnatal mammalian spinal cord
Source: Mol Neurodegener. 2017 Jan 19;12:8. doi: 10.1186/s13024-017-0148-1 (PMC5244531; doi:10.1186/s13024-017-0148-1)
Supplement: Additional file 1: Figure S1. — Sensory neurons exhibit neurodegenerative pathology without impaired nerve conduction in the absence of Gde2. This figure illustrates the presence of neuropathology in the primary sensory neurons of the Gde2 KO, including vacuolization, lipid accrual, and cytoskeletal accumulation; and the maintenance of peripheral sensory nerve conduction. Figure S2-related to Fig. 9. Conditional ablation of Gde2 in the postnatal spinal cord prevents the developmental loss of motor neurons. This figure confirms the effective conditional ablation of Gde2 following neurogenesis. In the constitutive KO, GDE2’s absence during embryonic neurogenesis causes a reduction of alpha motor neurons in the lateral motor column; however, in the Gde2lox/-; ROSA:CreER animals, this loss is avoided by injecting 4-OHT at E17.5. Further, competitive PCR analysis shows a near complete deletion of the conditional Gde2 allele following 4-OHT delivery. Figure S3. Gde2 deletion does not perturb neuromuscular junction morphology. This figure uses wholemount immunohistochemistry to assess the integrity of the neuromuscular junction (NMJ) in aged Gde2 KO hindlimb muscle. At 19 months, no discernible pathology is present in the Gde2 KO NMJ. (DOCX 6586 kb) [file 13024_2017_148_MOESM1_ESM.docx]

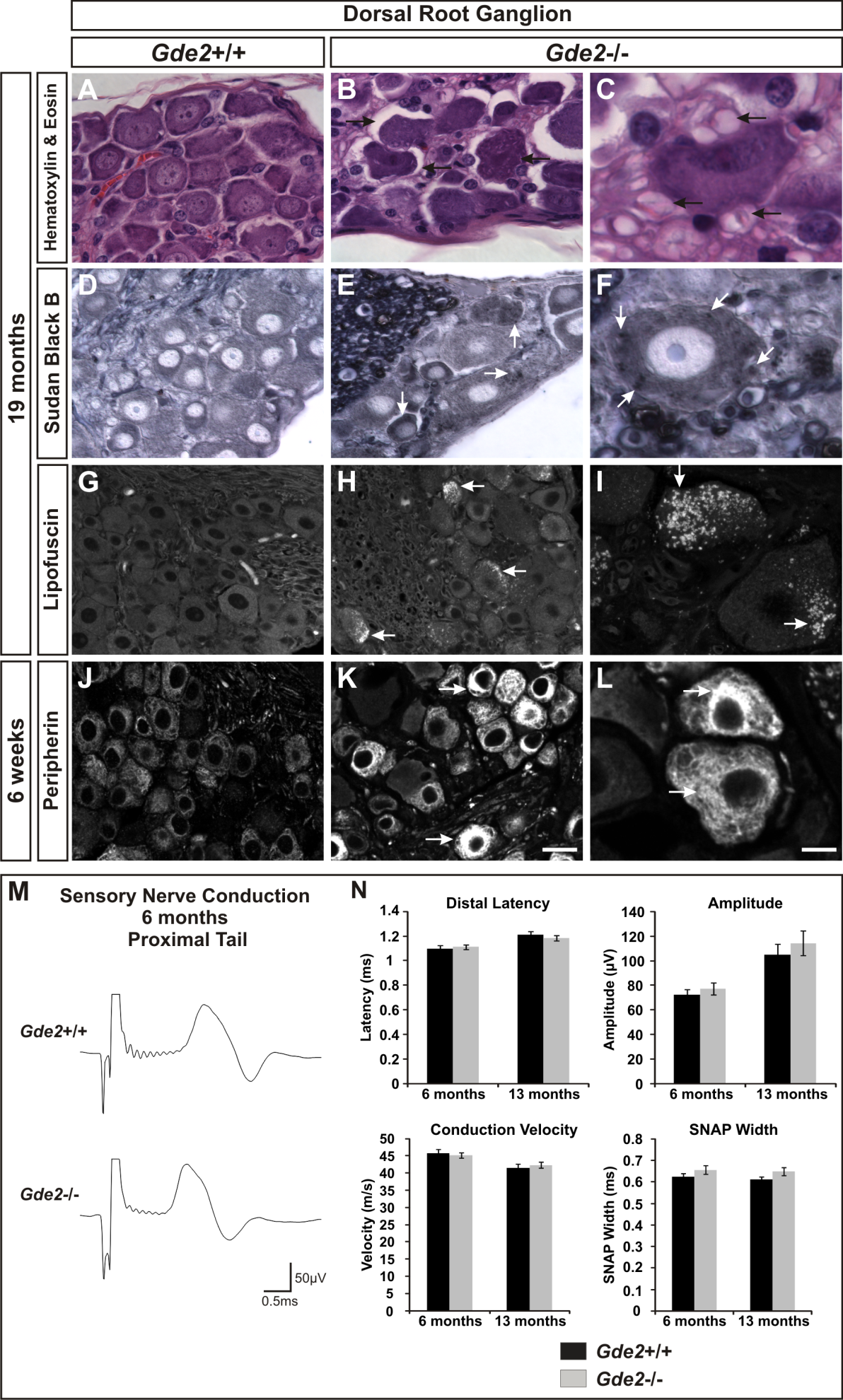


**Supplemental Figure 1. Sensory neurons exhibit neurodegenerative pathology without impaired nerve conduction in the absence of *Gde2***

(A-L) Transverse sections of paraffin embedded lumbar dorsal root ganglion (DRG) from WT and *Gde2* KO mice. (A-C) H&E staining shows vacuolization (arrows) in *Gde2* KO DRG neurons. (D-F) Sudan Black B staining highlights the accumulation of lipophilic puncta (arrows) in the *Gde2* KO. (G-H) *Gde2* KO DRG neurons accrue pathologic levels of autofluorescent lipofuscin puncta (arrows). (J-L). DRG neurons lacking *Gde2* exhibit enhanced immunoreactivity for cytoskeletal peripherin protein. Scale bar A,B,D,E,G,H,J,K = 15 μm. Scale bar C,F,I,L = 10 μm. n = 3. (M) Sensory Nerve Action Potential waveforms in WT and *Gde2* KO recorded from the proximal tail. (N) Graphs quantifying Distal Latency: 6 months p = 0.359, 13 months: 0.258; Amplitude: 6 months p = 0.228, 13 months p = 0.239; Conduction Velocity: 6 months p = 0.323, 13 months p = 0.279; SNAP Width: 6 months p = 0.117, 13 months p = 0.067, n = 10.


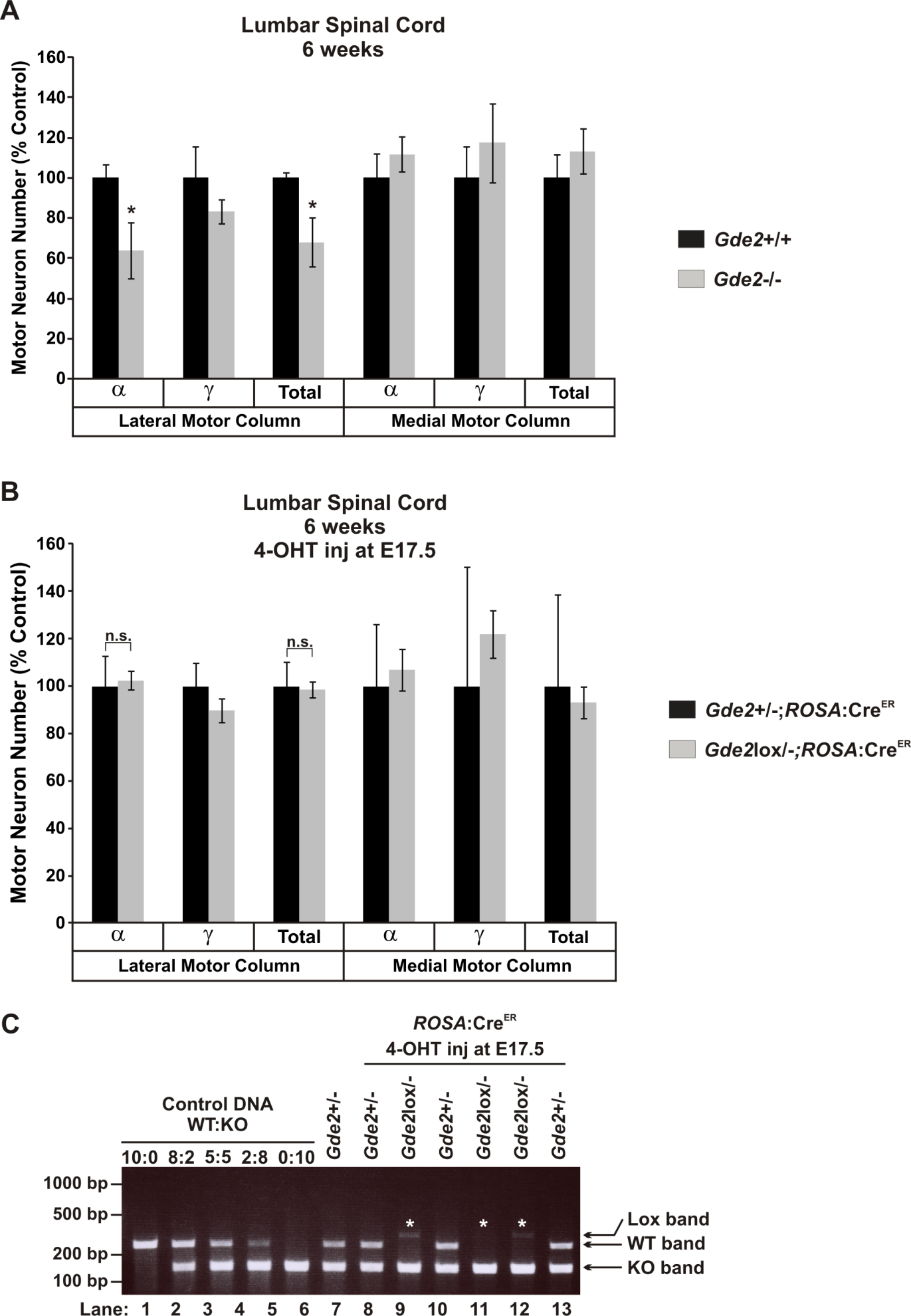


**Supplemental Figure 2-related to Figure 9. Conditional ablation of *Gde2* in the postnatal spinal cord prevents the developmental loss of motor neurons**

(A) Graph quantifying the motor neuron loss at 6 weeks resultant from the embryonic absence of *Gde2* in the constitutive *Gde2* KO. In the Lateral Motor Column (LMC), alpha motor neurons decrease by 36.03 ± 12.78% *p = 0.040, and total motor neurons are reduced by 27.49 ± 12.08% *p = 0.031. No changes are seen in the Medial Motor Column. (B) Postnatal removal of *Gde2*  via 4-Hydroxy-tamoxifen injections at E17.5 does not lead to motor neuron deficits at 6 weeks in either the LMC alpha p = 0.86 or total p = 0.90 motor neuron population in the *Gde2*^lox/-^ spinal cord. Graphs represent mean ± SEM, Student’s t test, n = 3. (C) Competitive PCR analysis visualizing the effective deletion of the *Gde2* lox allele following 4-OHT administration at E17.5. Lanes 1-6 show a gradient of WT and *Gde2* KO DNA mixed in the indicated proportions. Lane 7 is a *Gde2* heterozygote showing amplification of the WT and KO band. Lanes 8-13 show littermate *Gde2*+/-;*ROSA*Cre^ER^ and *Gde2*lox/-;*ROSA*Cre^ER^ mice. 4-OHT injection at E17.5 results in ≥ 80% reduction in the *Gde2* lox allele (asterisks) while having no affect on the WT allele.


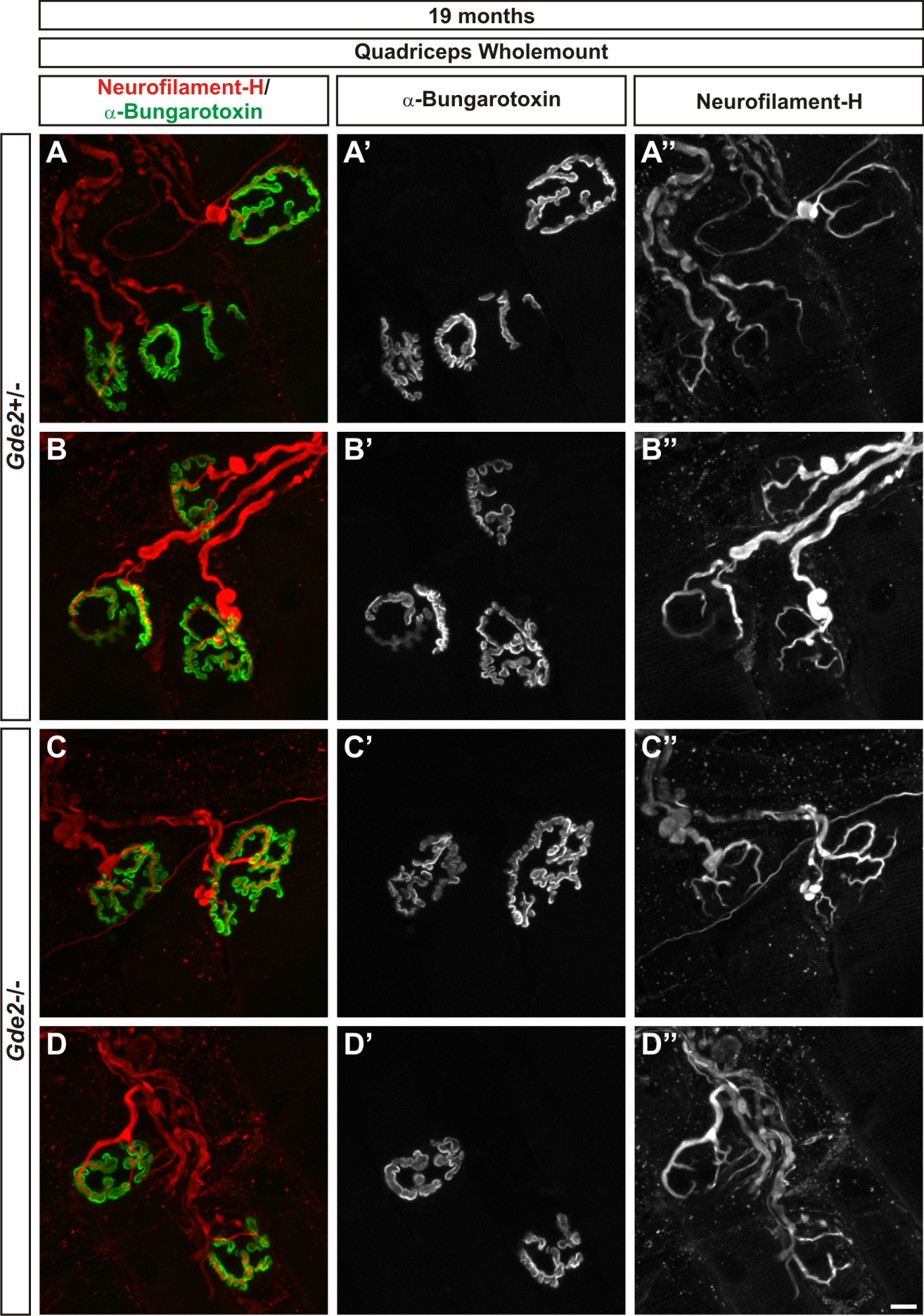


**Supplemental Figure 3. *Gde2* deletion does not perturb neuromuscular junction morphology**

(A-D’’) Confocal z-plane projections from quadriceps muscle wholemounts stained for Neurofilament-H and α-Bungarotoxin to visualize the pre- and postsynaptic components of the neuromuscular junction, respectively. 19 months *Gde2*+/- and *Gde2*-/- animals show equivalent neuromuscular junction morphology, n = 3. Scale bar = 10μm.
